# Neural Representation of Observed, Imagined, and Attempted Grasping Force in Motor Cortex of Individuals with Chronic Tetraplegia

**DOI:** 10.1038/s41598-020-58097-1

**Published:** 2020-01-29

**Authors:** Anisha Rastogi, Carlos E. Vargas-Irwin, Francis R. Willett, Jessica Abreu, Douglas C. Crowder, Brian A. Murphy, William D. Memberg, Jonathan P. Miller, Jennifer A. Sweet, Benjamin L. Walter, Sydney S. Cash, Paymon G. Rezaii, Brian Franco, Jad Saab, Sergey D. Stavisky, Krishna V. Shenoy, Jaimie M. Henderson, Leigh R. Hochberg, Robert F. Kirsch, A. Bolu Ajiboye

**Affiliations:** 1 Department of Biomedical Engineering, Case Western Reserve University, Cleveland, OH 44016 USA; 2 Department of Neuroscience, Brown University, Providence, RI 02912 USA; 3 Robert J. Nancy D. Carney Institute for Brain Sciences, Brown University, Providence, RI 02912 USA; 4 School of Engineering, Brown University, Providence, RI 02912 USA; 5 Neurosurgery, Stanford University, Stanford, CA 94035 USA; 6 Electrical Engineering, Stanford University, Stanford, CA 94035 USA; 7 Wu Tsai Neurosciences Institute, Stanford University, Stanford, CA 94035 USA; 8 Bioengineering, Stanford University, Stanford, CA 94035 USA; 9 Department of Neurobiology, Stanford University, Stanford, CA 94035 USA; 10 Howard Hughes Medical Institute, Stanford University, Stanford, CA 94035 USA; 11 Bio-X Program, Stanford University, Stanford, CA 94035 USA; 12 FES Center, Rehabilitation R&D Service, Louis Stokes Cleveland Department of VA Medical Center, Cleveland, OH 44016 USA; 13 Department of Neurological Surgery, UH Cleveland Med. Ctr., Cleveland, OH 44106 USA; 14 Department of Neurology, UH Cleveland Med. Ctr., Cleveland, OH 44106 USA; 15 Neurological Surgery, CWRU School of Medicine, Cleveland, OH 44106 USA; 16 Department of Neurology, Massachusetts General Hospital, Boston, MA 02114 USA; 17 Center for Neurotechnology and Neurorecovery, Department of Neurology, Massachusetts General Hospital, Boston, MA 02114 USA; 18 VA RR&D Center for Neurorestoration and Neurotechnology, Department of VA Medical Center, Providence, RI 02912 USA; 19 Department of Neurology, Harvard Medical School, Boston, MA 02115 USA

**Keywords:** Brain-machine interface, Neurology, Biomedical engineering

## Abstract

Hybrid kinetic and kinematic intracortical brain-computer interfaces (iBCIs) have the potential to restore functional grasping and object interaction capabilities in individuals with tetraplegia. This requires an understanding of how kinetic information is represented in neural activity, and how this representation is affected by non-motor parameters such as *volitional state* (VoS), namely, whether one observes, imagines, or attempts an action. To this end, this work investigates how motor cortical neural activity changes when three human participants with tetraplegia *observe, imagine*, and *attempt* to produce three discrete hand grasping forces with the dominant hand. We show that force representation follows the same VoS-related trends as previously shown for directional arm movements; namely, that attempted force production recruits more neural activity compared to observed or imagined force production. Additionally, VoS-modulated neural activity to a greater extent than grasping force. Neural representation of forces was lower than expected, possibly due to compromised somatosensory pathways in individuals with tetraplegia, which have been shown to influence motor cortical activity. Nevertheless, attempted forces (but not always observed or imagined forces) could be decoded significantly above chance, thereby potentially providing relevant information towards the development of a hybrid kinetic and kinematic iBCI.

## Introduction

Intracortical brain-computer interfaces (iBCIs) that command neuroprosthetics have the potential to restore lost or compromised function to individuals with tetraplegia. iBCIs typically detect neural activity from motor cortex, which encodes kinetic and kinematic information in rhesus macaques^1–14^. iBCIs that extract kinematic parameters have allowed individuals to command one- and two-dimensional computer cursors^15–26^, prosthetics^27–29^, and functional electrical stimulation of paralyzed muscles^30,31^. Additional work has characterized closed-loop kinetic control in nonhuman primates^32–34^ and open-loop force modulation in human participants^35,36^. These studies could potentially move iBCI technology towards restoring functional tasks requiring both kinetic and kinematic control.

Motor cortex can exhibit activity in the absence of motor output, such as during mental rehearsal or movement observation^37,38^. Furthermore, rather than representing fixed motor parameters, M1 may adapt to achieve the task at hand^39^. A body of work has investigated how motor cortex modulates to different *volitional states*, including passive observation, imagined action, and attempted or executed action, mostly in the context of kinematic outputs. For example, kinematic imagery produces similar neural activity patterns as movement execution – or attempted movement, in persons with tetraplegia – but more weakly and in a smaller subset of motor areas^40–43^. Furthermore, in individuals with tetraplegia, observed, imagined, and attempted arm reaches recruit shared neural populations, but nonetheless yield unique patterns of activity^44^. This supports the existence of a “core” network that modulates to all volitional states, and the recruitment of additional neural circuitry during the progression from passive observation to attempted movement^44–48^.

The effects of volitional state on non-kinematic representation, including that of grasping forces, remains uncertain. In able-bodied participants with implanted with sEEG electrodes, executed forces produce stronger neural signals than imagined forces^49^. This supports fMRI findings in able-bodied individuals and participants with spinal cord injury, who exhibited weaker, less widespread BOLD activations when imagining (vs. attempting) forces^50^. However, while this trend was readily apparent during a standard two-tailed analysis of all able-bodied subjects, it only appeared in the SCI group when within-subject comparisons were implemented.

To our knowledge, neural modulation to observed, imagined, and attempted forces has not been evaluated at the resolution of intracortical activity in humans. Furthermore, while open-loop force decoding was achieved in a single individual with tetraplegia^36^, the extent to which force is neurally represented in this patient population, or how volitional state may affect this representation, is unresolved. Therefore, the present study evaluates how volitional state (observe, imagine, and attempt) during kinetic behavior (grasp force production) influences neural activity in motor cortex. Specifically, we characterize the topography of the neural space representing force and volitional state in three individuals with tetraplegia, both at the level of single neural features extracted from multiunit intracortical activity, and at the level of the neural population. We show that 1) volitional state affects how neural activity modulates to force; namely, that attempted forces generate stronger cortical modulation than observed and imagined forces; 2) grasping forces are reliably decoded when attempted, but not always when observed or imagined, and 3) volitional state is represented to a greater degree than grasping forces in the motor cortex.

## Results

### Characterization of individual features

**Figure 1:**
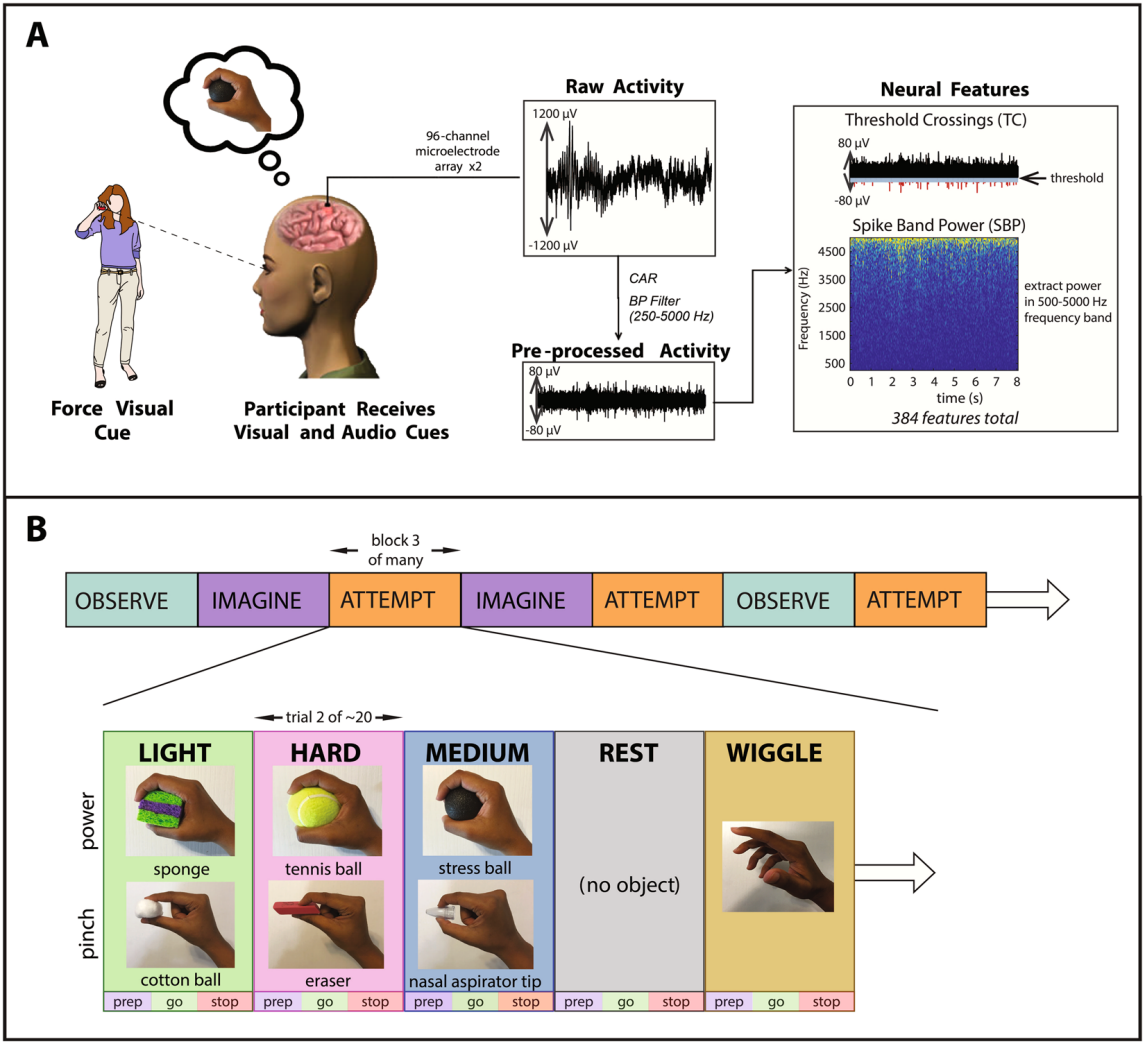
(**A**) Experimental setup. Prior to the current study, participants were implanted with two 96-channel microelectrode arrays in motor cortex as a part of the BrainGate2 Pilot Clinical Trial. The microelectrode arrays recorded neural activity while participants completed a force task. Two neural features (threshold crossings, spike band powers) were extracted from each channel in the arrays. (**B**) Experimental session architecture. Each session consisted of 12–21 blocks, each of which contained ~20 trials (see Supplementary Table S1). In each trial, participants observed, imagined, or attempted to generate one of three cued forces with either a power grasp or a closed pincer grasp; to perform a wiggling finger movement; or to rest. Trial types were presented in a pseudorandom order. Each trial contained a preparatory (prep) phase, a go phase where forces were actively embodied, and a stop phase where neural activity was allowed to return to baseline. Participants were prompted with both audio and visual cues, in which a researcher squeezed an object associated with each force level. Visual cues were presented with a third person, frontal view, in which the researcher faced the participants while squeezing the objects. Lateral views are shown here for visual clarity, but were not displayed as such to the participants.

A major goal of this study was to determine whether force-related tuning was present at the level of single neural features extracted from multiunit intracortical activity (Fig. 1A), and to assess the extent to which volitional state affected this tuning. These neural features were extracted during a force-matching behavioral task, as illustrated in Fig. 1B, in which participants T8, T5, and T9 *observed, imagined*, and *attempted* producing three discrete forces using either a power or a pincer grasp. Supplementary Table S1 shows a full list of sessions and their associated parameters. In all participants, two neural features were extracted from each of the 192 recording electrodes implanted in motor cortex. Threshold crossing (TC) features, defined as the number of times the neural activity crossed a pre-defined, channel-specific noise threshold, are numbered from 1–192 according to the recording electrode from which they originate. Corresponding spike band power (SBP) features, defined as the root mean square of the signal in the spike band (250–5000 Hz) of each channel, are numbered from 193–384.

**Figure 2:**
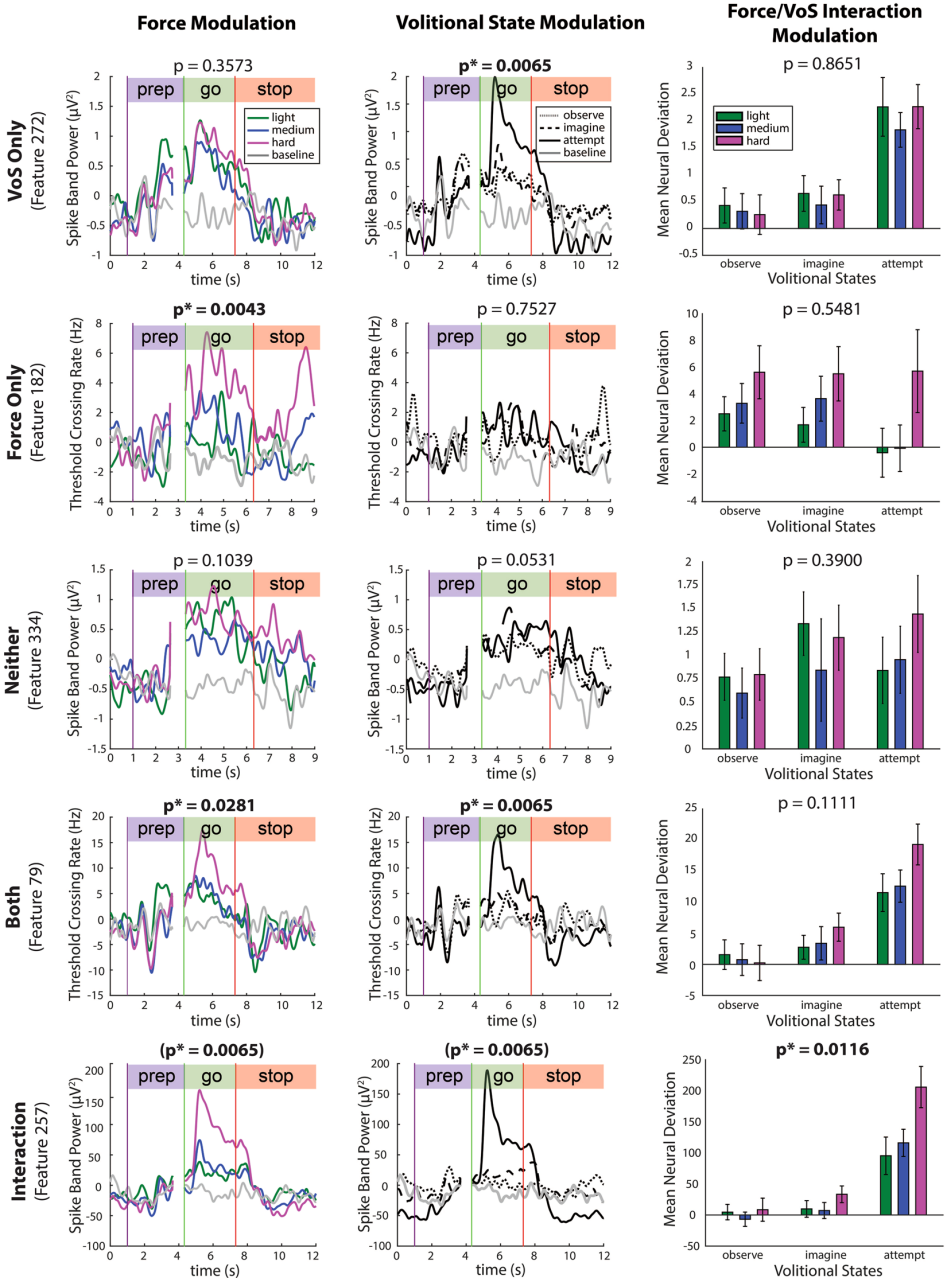
Single features are tuned to force and volitional state. Rows: Average per-condition activity (PSTH) of five exemplary TC and SBP features tuned to force only (session 1), volitional state (VoS) only (session 4), neither factor (session 1), both factors (session 4), and an interaction between both factors (session 4) in participant T8 (2-way Welch ANOVA, corrected p < 0.05, Benjamini-Hochberg method). Neural activity was smoothed with a 100-ms Gaussian kernel prior to trial averaging to aid in visualization. Statistically significant p-values for force modulation, VoS modulation, and interaction are indicated with asterisks. Neural activity in Column 1 is averaged over all volitional states, such that observable differences in modulation are due to force alone (~50–90 trials per force level, depending on session number). Similarly, Column 2 depicts the activity of individual features during distinct volitional states, averaged over all force levels (~50–90 trials per volitional state, depending on session number). Simple main effects are represented graphically in Column 3 via normalized mean neural deviations from baseline activity during force trials within each of the three volitional states. Modulation depths were computed over the go phase of each trial, and then averaged within each force-VoS pair. Error bars indicate 95% confidence intervals.

Figure 2 shows four representative TC and SBP features in participant T8 that are tuned to one of four marginalizations of parameters as evaluated with 2-way Welch-ANOVA: *force only*, *volitional state only*, *neither* force nor volitional state, *both* force and volitional state independently, and an *interaction* between force and volitional state. Supplementary Figs. S6 and S7 show corresponding feature traces for participants T5 and T9, respectively. Additionally, Supplementary Figs. S8–S11 contain rasters for exemplary single sorted units that are tuned to these factors in participant T8.

For each neural feature, peristimulus time histograms (PSTHs) averaged within individual forces (Column 1) and within individual volitional states (Column 2) are illustrated in Fig. 2. SBP Feature 272 was tuned to *force only* (row 2); as such, it showed go-phase differentiation across multiple force levels that were statistically discriminable (corrected p < 0.05, 2-way Welch-ANOVA, Benjamini-Hochberg method). In contrast, TC feature 182 was tuned to *volitional state only* (row 1), and, therefore, did not exhibit force discriminability but were discriminable across multiple volitional states (corrected p < 0.05, 2-way Welch-ANOVA). TC Feature 79, which was tuned independently to *both* force and volitional state, was statistically discriminable for both parameters.

Column 3 of Fig. 2 graphically represents the simple main effects of the 2-way Welch-ANOVA analysis, as represented by mean go-phase neural deviations from baseline activity for each force level within each volitional state. In these panels, features that are tuned independently to force exhibit a similar pattern of modulation to light, medium, and hard grasping forces, regardless of the volitional state used in the trial. These include TC Feature 182 (row 2), which is tuned to force only, and TC Feature 79 (row 4), which is tuned independently to both force and volitional state. In contrast, SBP Feature 257 (row 5) exhibits a statistically significant interaction between force and volitional state (p < 0.05). For this feature, the mean neural deviations attributed to each force level are affected by the volitional states used to emulate them. This suggests that volitional state could affect the degree to which interacting features are tuned to force in participant T8.

**Figure 3:**
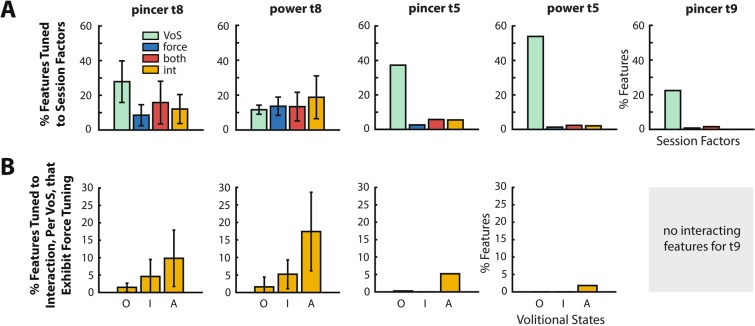
Overall Tuning of Neural Features. (**A**) Fraction of neural features significantly tuned (2-way Welch-ANOVA, corrected p < 0.05) to force and/or volitional state during the go phase of force production. For participant T8, results are averaged across multiple sessions. Error bars indicate standard deviation. (**B**) Fraction of total features exhibiting a statistically significant interaction (2-way Welch-ANOVA, p < 0.05) between force and volitional state, subdivided into force-tuned features during observation (O), imagination (I), and attempt (A). Force tuning within each volitional state was determined via one-way Welch-ANOVA (corrected p < 0.05). Error bars indicate standard deviation. Results show that features with an interaction between force and VoS are more likely to have force tuning in the attempt condition than in the observed or imagined conditions.

Figure 3 summarizes the tuning of all 384 TC and SBP features in each participant across both power and pincer grasps, during the active “go” phase of the behavioral task. In participant T8, these data are averaged over multiple experimental sessions. Figure 3A shows the average fraction of features in the neural population that were tuned to the four marginalizations of interest (corrected p < 0.05, 2-way Welch-ANOVA). In all participants, a substantial proportion of modulating features are tuned to volitional state only. A second population of features – denoted by blue and red bars – exhibit independent go-phase tuning to force (27.0% in T8 power, 24.3% in T8 pincer, 3.6% in T5 power, 8.3% in T5 pincer, and 2.3% in T9 pincer). A majority of these force-tuned features exhibit “mixed selectivity”, in that they are also independently tuned to volitional state. A final subset of features exhibits a statistically significant interaction (corrected p < 0.05, 2-way Welch-ANOVA) between force-related and volitional-state-related modulation. Figure 3B further subdivides these interacting features into those that are tuned to force within each individual volitional state (corrected p < 0.05, 1-way Welch ANOVA). Note that in participant T9, no interacting features were detected. In participants T8 and T5, a larger proportion of interacting features are recruited by attempted forces than by observed and imagined forces. Therefore, in these two participants, an overall greater proportion of neural features are tuned to force during the attempted volitional state.

### Neural population analyses

The next goal was to examine how volitional state affected force-related tuning at the neural population level. To this end, feature vectors representing individual trials were projected into low dimensional “similarity spaces” using continuous similarity (CSIM) analysis, adapted from Spike-Train Similarity (SSIM) analysis^51^, as described in the Methods. Briefly, CSIM makes pair-wise comparisons between patterns in neural features and then projects these pair-wise comparisons into a low-dimensional representation using t-distributed Stochastic Neighbor Embedding (t-SNE)^52^. In the low-dimensional representation, feature activity during an individual trial is represented as a single point, and the distance between points denotes the degree of similarity between trials.

**Figure 4:**
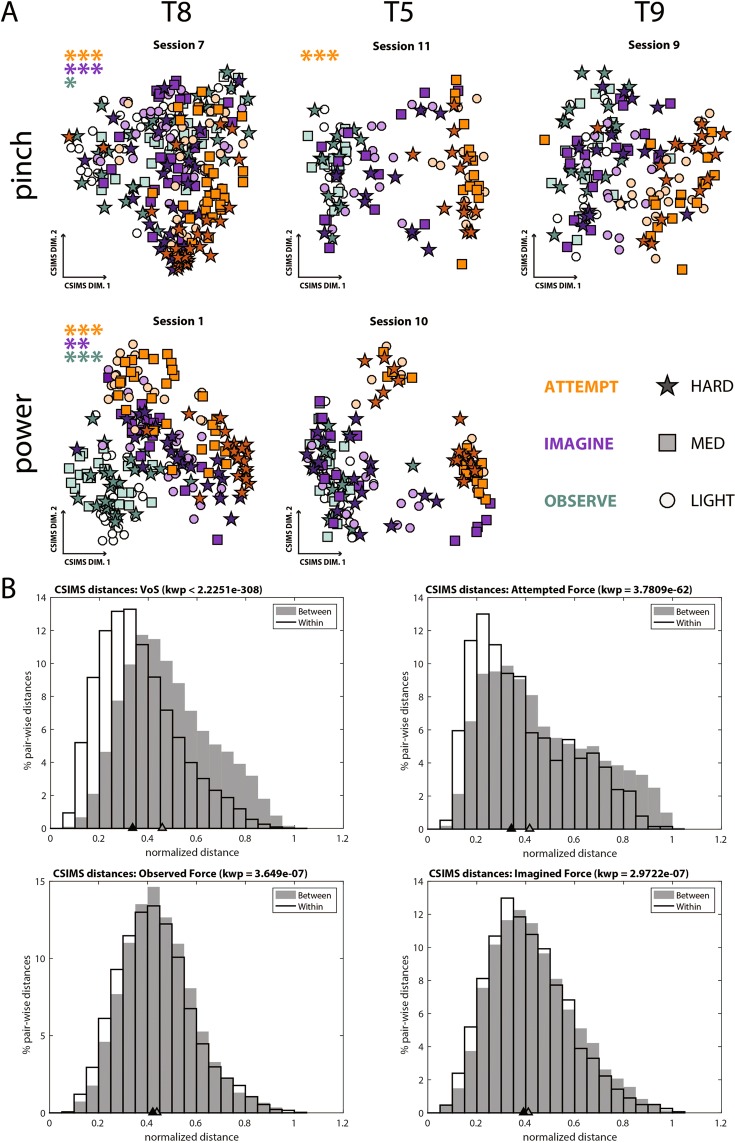
Feature population activity patterns. (**A**) Two-dimensional CSIM plots for a representative session from each participant-grasp pair. Each point represents the activity of the entire population of simultaneously-extracted features during a single trial. The distance between points indicates the degree of similarity between single trials. Clustering of similar symbols denotes similarity between trials with the same intended force level, while clustering of similar colors indicates similarity of trials within the same volitional state. In all panels, the distribution of distances for pairs of trials within the same VoS displayed a significantly smaller median than distances between trials in different VoS categories (Kruskall-Wallis p < 0.00001). Analyzing pair-wise distances for trials within and between force conditions produced different results across sessions. Asterisks in the top left corner denote sessions with significantly smaller within-force than across-force distances (*p < 0.05 **p < 0.01, and ***p < 0.0001) within each VoS, as indicated by the color of the asterisks. (**B**) Distribution of pairwise distances within and between categories for VoS (upper left) and and observed, imagined, and attempted force. Distances were normalized and pooled across all sessions shown. Triangles on the X-axis denote medians for each distribution. Overall, VoS had a stronger effect on trial similarity than even the attempted force condition.

Figure 4A shows the relationship between single-trial activity patterns using two-dimensional CSIM plots for a representative session from each participant-grasp pair. In concordance with the single feature data, clustering of trials according to volitional state is evident in all participants during both power and pincer grasping (Kruskall-Wallis p < 0.00001). In contrast, force-related clustering of trials occurred only during sessions 1, 7, and 11 and was most apparent during attempted force production (Kruskal-Wallis, p < 0.0001). These trends are further illustrated in Fig. 4B, which shows the distribution of pairwise distances between trials within and between volitional states in the upper left panel, as well as within and between forces within each volitional state. The medians corresponding to the within-condition and between-condition distributions are farthest apart for volitional state, are closest together for observed and imagined forces, and are separated by an intermediate amount for attempted forces. This indicates that volitional state has a stronger influence on trial similarity than even attempted forces. In other words, trials cluster more readily according to volitional state than to force in the CSIM space.

**Figure 5:**
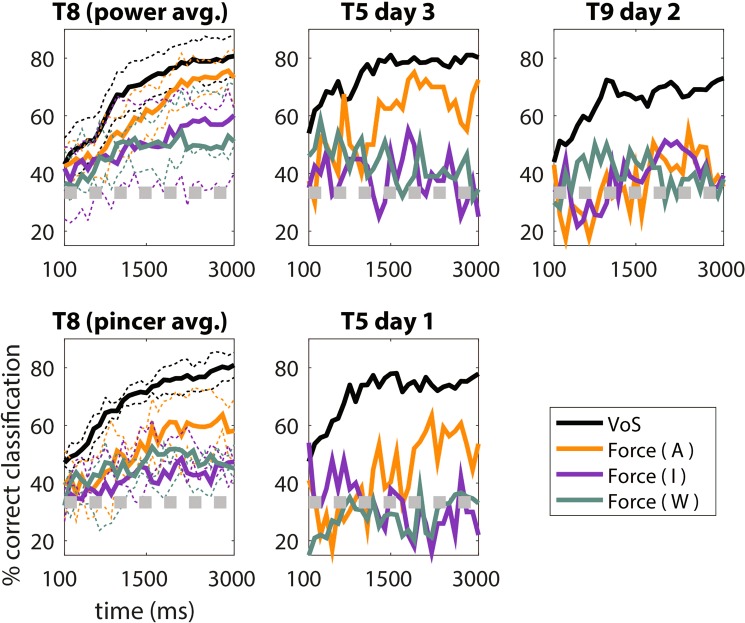
Feature ensemble CSIM force and volitional state decoding accuracies as a function of window length. Offline decoding accuracies were computed using an LDA classifier implemented within a 10-dimensional CSIM representation of the neural feature data, using 10-fold cross-validation. 10-dimensional CSIM data of window lengths ranging from 100 ms to 3000 ms were passed to the LDA, as described in the Methods. Each window began at the start of the go phase and ended at the time point indicated on the x-axis. For participant T8, each panel shows session-averaged decoding performances from each participant-grasp pair. The T8 power and pincer panels were averaged over 5 and 3 sessions, respectively. Standard deviations across T8 sessions are indicated by the dotted lines. Gray line indicates the upper boundary of the 95% empirical confidence interval of the chance distribution, estimated using 10,000 random shuffles of the trial labels.

**Figure 6:**
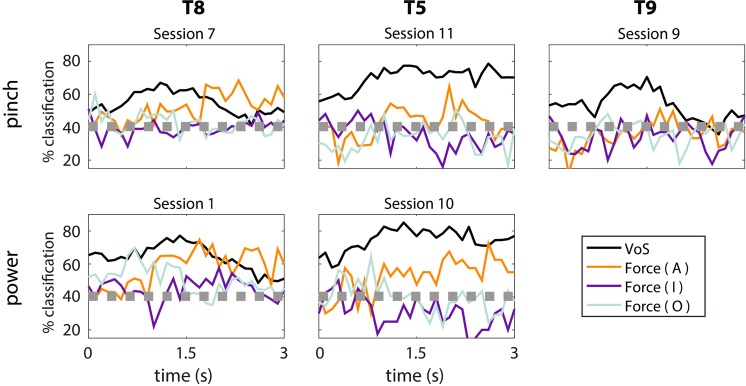
Time-dependent feature ensemble CSIM force and volitional state decoding accuracies. Offline decoding accuracies were computed using an LDA classifier implemented within a 10-dimensional CSIM representation of the neural feature data, using 10-fold cross-validation. The LDA was applied to a 400 ms sliding window, stepped in 100 ms increments. Each panel shows decoding performance for a representative session from each participant-grasp pair, where time = 0 indicates the start of the active “go” phase of the trial. The gray line indicates the upper boundary of the 95% empirical confidence interval of the chance distribution, estimated using 10,000 random shuffles of the trial labels.

In order to further quantify the degree to which volitional state and force were separable at the population level, VoS and force classification was performed using an LDA classifier operating on 10-dimensional CSIM space projections. To determine the time resolution at which force- and VoS-related information could be decoded, the LDA was applied to 10-dimensional CSIM data of varying window lengths, beginning at the start of the go phase, as described in the Methods and as shown in Fig. 5. Further, to determine how force and volitional state were represented in the neural space as a function of time, time-dependent classification accuracies were determined by applying the LDA to CSIM data with a 400 millisecond sliding window stepped in 100 millisecond increments (Fig. 6). For both of these analyses, chance performance was estimated by applying the LDA to the neural data during 10,000 random shuffles of the trial labels. The mean of the empirical chance distribution (averaged across participants) was 33.9%, with 95% of samples between 26.6 and 40.3%. Both Figs. 5 and 6 show that, in agreement with the structure observed in the CSIM neural space in Fig. 4, VoS is decoded with greater accuracy than force for all participants and grasps. However, force-related information also appears to be present, and is decoded above chance throughout the go phase of attempted force production in all participants (but not always across other VoS conditions). In other words, volitional state appears to affect the degree to which force is represented at the level of the neural population.

**Figure 7:**
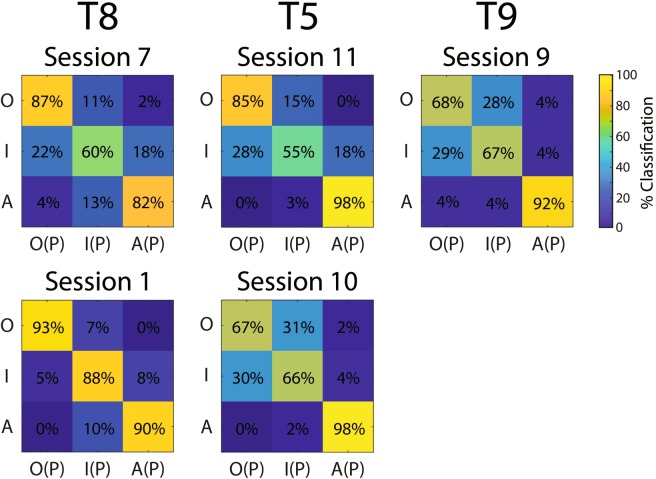
Feature ensemble volitional state go-phase confusion matrices. Offline decoding accuracies were computed using an LDA classifier implemented within a 10-dimensional CSIM representation of the neural feature data, using 10-fold cross-validation, over a 400 ms sliding window stepped down in 100 ms increments. Classification accuracies for individual volitional states were averaged over all time points within the go phase of the trial, resulting in a confusion matrices of true vs. predicted (P) observed (O), imagined (I), and attempted (A) volitional states. Note that the attempted volitional state is classified with a high accuracy rate across all sessions, while observed trials are classified with high accuracy during sessions 1, 7, and 11.

To elaborate the extent to which individual volitional states were represented in the neural space, classification accuracies of individual volitional states were computed, averaged over the go phase of the behavioral task, and then compiled into confusion matrices. Figure 7 shows that, while all volitional states are classified at above-chance accuracy, observation and attempt appear to be classified with greater accuracy than imagery in multiple datasets. In particular, attempt is classified with high accuracy across all sessions, while observation is classified with high accuracy during Sessions 1, 7, and 11. During Sessions 9 and 10, observation and imagery are classified with similar accuracy and tend to be confused with each other more often than they are confused with attempt. These results suggest that attempt (and possibly observation) drives volitional state representation to a greater degree than imagery.

## Discussion

### Force representation persists in motor cortex after tetraplegia

The primary study goal was to characterize how volitional state affects neural representation of force in human motor cortex at the feature-ensemble level. We found that force-related activity persists in motor cortex, even after tetraplegia. This validates fMRI findings in individuals with spinal cord injury, who exhibited BOLD activity that modulated to imagined force^50^; as well as intracortical findings in an individual with tetraplegia^36^. The present work expands upon these studies by demonstrating force-related activity in multiple people with paralysis within single features (Figs. 2–3) and the neural population (Figs. 4–6).

The present work also shows quantitative, electrophysiological evidence that force modulation depends on volitional state. Briefly, attempted forces recruit more single features (Fig. 3), separate more distinctly in the CSIMS neural space (Fig. 4), and yield higher classification accuracies (Figs. 5–6) than observed and imagined forces. This validates previous kinematic^40–44^ and kinetic^49,50^ volitional state studies, in which attempted actions were more readily decoded and yielded stronger BOLD activations than other volitional states.

### Volitional state modulates neural activity to a greater extent than force

The present work demonstrates that volitional state is represented robustly within the neural space in individuals with tetraplegia (Figs. 4–7). In particular, individual volitional states recruit an overlapping population of neural features (Fig. 3), but nonetheless result in unique activity patterns (Fig. 4). This supports the theory of overlapping yet distinct neural populations corresponding to each volitional state^44^. Additionally, while individual volitional states are represented at above-chance levels in all participants, the attempted state recruits the most neural features (Fig. 3) and is classified with high accuracy (Fig. 7), compared to the other volitional states. In contrast, the imagined volitional state was classified least accurately and was often confused with observation. These results appear to be consistent with previous intracortical volitional state investigations in humans, in which the attempted state was shown to recruit more single units and result in higher firing rates, while the imagined state recruited the fewest number of single units in a human participant with the highest neural volitional state representation^44^. Taken together, the current study results suggest that volitional states are represented similarly in multiple individuals with tetraplegia, regardless of whether these states were used to emulate kinematic vs. kinetic tasks.

Here, however, force representation was weaker than volitional state representation. Specifically, while many features modulated primarily to volitional state, few exhibited force tuning; and most of these were also tuned to volitional state (Fig. 3). Additionally, volitional state was discriminated more reliably from feature ensemble activity than even attempted forces (Figs. 4–6). This relative deficiency of force information, which contrasts with previous work in nonhuman primates^1–3,12,53^ and able-bodied humans^35,49,50,54–57^, could have potentially resulted from several factors. Here, we discuss the effects of two such factors, which include the visual cues used to prompt the force task and the effects of deafferentation in individuals with tetraplegia.

#### Effects of visual cues on neural activity

During most experimental sessions presented in this study, visual cues were used to prompt the observation, imagery, and attempt of forces. Briefly, a researcher lifted one of six graspable objects associated corresponding to light, medium, and hard power and pincer grasping forces to indicate the preparatory phase; squeezed the object during the active “go” phase; and ultimately released the object at the beginning of the stop phase of the trial. In response to these visual cues, participants were instructed to observe, imagine, or attempt emulating sufficient force to crush the object squeezed by the researcher.

As discussed, this visually-cued behavioral task yielded three separate yet overlapping populations of the neural features corresponding to each volitional state. Previous studies in non-human primates have identified a class of cells within motor cortex, termed “mirror neurons”, that exhibit changes in activity in response to the observed and executed volitional states^58–61^. The presence of these neurons may account for the degree of overlap between feature populations tuned to observation, imagery, and attempt. Additional studies have demonstrated that the activity of neurons responding to observed and executed actions depends on characteristics of how the motor actions are observed. For example, neural activity has been shown to depend on whether the observed action is static versus dynamic^62^, whether it occurs within versus beyond the participant’s reach^63,64^, and whether it is presented within an allocentric versus an egocentric reference frame^65,66^.

The visual cues in this study were static during the active “go” phase of the task, occurred within the participants’ extrapersonal space, and were presented using a third-person perspective. Since some of these characteristics are associated with weaker neural activity during action observation^62,63,65^, the visual cues included in this study may partially account for the weak force representation demonstrated here. However, many of these same non-human primate studies suggest that static, third-person, and extrapersonal visual cues can still elicit robust neural responses to observed actions in able-bodied individuals. For example, one study demonstrated force encoding during the reach phase of an observed motor task–before contact had been made with the object, such that the only force-related information available to the monkey was from previous knowledge of the object’s weight^67^. In light of these findings, it is likely that robust force-related representation can be elicited even in response to relatively “weak” visual stimuli, such as the static squeezing of graspable objects. Furthermore, while separate yet overlapping populations of neurons respond to first-person versus third-person views of motor acts, the number of neurons within each of these populations is relatively similar, albeit larger for first-person views^65^. Similar results were found for neurons recruited during the observation of motor acts within the peripersonal versus the extrapersonal space^63^. Therefore, while it is still possible that the current study’s visual cues contributed to the weak force representation observed here, this contribution was likely minor.

#### Effects of deafferentation in individuals with tetraplegia

An additional explanation for the deficiency in neural force representation could include the effects of deafferentation-induced cortical reorganization in tetraplegia^68,69^. Notably, these effects were absent in the previously cited non-human primate literature, which predicts robust representation of observed and executed motor actions in able-bodied individuals^63,65,67^. In individuals with spinal cord injury, force-related BOLD activity has been shown to overlap minimally with able-bodied force activity^50^, suggesting that altered cortical networks in tetraplegia could affect the extent of force representation in motor cortex. However, kinematic representation is well preserved in tetraplegia, as evidenced by high decoding accuracies in kinematic iBCIs^16,18,19,23,27–30,44^. Therefore, a discrepancy exists between kinematic and kinetic representation in tetraplegia.

This discrepancy may result from sensory feedback differences between the able-bodied and paralyzed states – and indeed, between kinematic and kinetic tasks attempted by individuals with tetraplegia. Without sensory feedback, motor performance is significantly compromised^70–72^; while reintroducing visual^56,70^, auditory^57^, tactile^73^, or proprioceptive^74^ feedback enhances motor-related neural modulation and BCI control. Moreover, in an individual with tetraplegia who had intact sensation, motor cortical neurons modulated to both passive joint manipulation and attempted arm actions^75^. Taken together, these studies suggest that multiple sensory inputs influence motor cortical activity, and that diminished sensory feedback compromises modulation to motor parameters.

Of all sensory modalities, tactile and proprioceptive feedback are the most relevant to fine control of grasping forces^73,76,77^. This is because kinetic tasks are largely controlled through feedback via somatosensory pathways^78,79^, which are profoundly altered in tetraplegia^80^. Participants T8, T5, and T9 were deafferented and received no feedback regarding their own force output. In contrast, kinematic studies in people with paralysis were performed in the context of directional movements that rely on visual feedback, which remains intact after tetraplegia. After complete sensorimotor deafferentation, parameters relying on visual pathways could be preserved in motor cortex, while those relying on somatosensory feedback could diminish. This could explain the relatively weak force tuning observed here, as opposed to the robust force modulation seen in able-bodied literature.

### Limitations of open-loop task

In this study, participants observed, imagined, and attempted discrete grasping forces, with the understanding that they could not execute these forces. This open-loop investigation is a key first step towards elucidating neural force representation; however, it came with inherent limitations regarding how participants engaged in the task. For example, volitional states may be challenging for some individuals with paralysis to differentiate cognitively, though previous work suggests this is generally achievable^44^. Additionally, since participants received no sensory feedback, the study findings depended on each participant’s understanding of the task and their ability to kinesthetically observe/imagine/attempt discrete force levels despite their chronic tetraplegia. Furthermore, while the use of visual cues enhanced this understanding by increasing the applicability of the force task to participants, the various objects and the hand shapes used to squeeze them may have been reflected in the neural data along with forces and volitional states. Finally, while participants were instructed to vary only the amount of force needed to crush these objects, there was no way to measure their intended kinematic and kinetic outputs, which could have differed somewhat between force levels.

To address and mitigate these challenges, participants completed a Kinesthetic Force Imagery Questionnaire (KFIQ), adapted from the Kinesthetic and Visual Imagery Questionnaire^81^, in order to qualitatively assess the degree to which they emulated various force levels during each volitional state. In addition, participants reported in their own words how they differentiated between observed, imagined, and attempted forces. For example, participant T8 reported conceiving of a virtual arm performing the task during force imagery, whereas he emulated forces with his own arm during the attempt state. In all participants, KFIQ scores rose with volitional state in concordance with the neural data, as shown in Supplementary Fig. S5.

Furthermore, prior to completing the sessions presented in this main text, participant T8 completed a series of additional sessions, summarized in Supplementary Table S2, in which he was asked to observe, imagine, and attempt producing forces both when visual cues were included and when visual cues were omitted. Supplementary Figs. S1 and S2 show that the visual cues do introduce extraneous preparatory and stop phase neural activity within force trials, which is in agreement with previous non-human primate studies of neural activity occurring immediately before and after observed force tasks^60,82^. However, Supplementary Figs. S2 and S3 suggest that the visual cues do not influence neural activity during the active “go” phase of the trial.

Finally, participants performed kinematic control trials where they embodied finger wiggling movements. Supplementary Fig. S4 shows that force trials were more correlated with each other than with kinematic trials, especially during attempted forces. Additionally, the participants’ KFIQ scores, which reflect embodiment of forces as opposed to kinematics, were often lower for finger wiggling than for force (Supplementary Fig. S5). Though only a closed-loop study could guarantee that participants were emulating forces, the KFIQ scores, and their correlation with the neural data, indicate that this was likely the case.

### Force representation differences across participants

Relative to other participants, T8’s motor cortical activity exhibited the most force-tuned features, the clearest separation between attempted forces in CSIM space, and the highest force decoding accuracy. In contrast, T5’s force modulation was more robust during pincer grasping (Session 11) than during power grasping (Session 10), and T9’s force modulation appeared to be relatively weak overall. The study’s open-loop nature partially explains these discrepancies: T5 had difficulty distinguishing light, medium, and hard forces during Session 10, but improved during Session 11. Additionally, differences in pathology may account for T9’s relatively weak force representation. While T8 and T5 were otherwise neurologically healthy participants with cervical spinal cord injury, T9 had advanced ALS, which may have comparatively affected his motor-related cortical activity. Future work with a greater number of participants, with different paralysis etiologies, will be needed to more comprehensively investigate these inter-participant differences.

### Implications for iBCI development

A long-term motivation behind this study is to investigate the feasibility of incorporating force control into closed-loop iBCIs. This work validates the presence of kinetic information in motor cortex and shows that volitional state influences force modulation. Therefore, while decoding intended forces in real time appears feasible, future closed-loop force iBCIs will need to take non-motor parameters into account. For example, the data suggest that iBCI force decoders should be trained on neural activity generated during attempted (as opposed to imagined) force production.

More broadly, accurate force decoding will likely depend on increasing neural force representation in individuals with tetraplegia. Since closed-loop decoding performance often exceeds open-loop performance^83–85^, simply providing visual feedback about intended forces could improve neural force representation. In addition, somatosensory feedback has been shown to elicit motor cortical activity in a tetraplegic individual with intact sensation^75^. This supports the possibility of enhancing force-related neural representation with somatosensory feedback. In completely deafferented individuals, graded tactile percepts have been evoked on the hand via microstimulation of somatosensory cortex^77,86^, indicating that it is possible to provide closed-loop sensory feedback about intended grasping forces in this population. More work is needed to determine the extent to which sensory feedback affects motor cortical force representation, and how this representation translates into closed-loop iBCI force decoding performance. Nevertheless, this study shows promise for informing future closed loop iBCI design and provides further insight into to the complexity of motor cortex.

## Methods

### Study permissions and participants

Study procedures were approved by the US Food and Drug Administration (Investigational Device Exemption #G090003) and the Institutional Review Boards of University Hospitals Case Medical Center (protocol #04-12-17), Massachusetts General Hospital (2011P001036), the Providence VA Medical Center (2011-009), Brown University (0809992560), and Stanford University (protocol #20804). Participants were enrolled in the BrainGate2 Pilot Clinical Trial (ClinicalTrials.gov number NCT00912041). All research was performed in accordance with relevant guidelines and regulations. Informed consent, including consent to publish, was obtained from the participants prior to their enrollment in the study.

This study includes data from three participants with chronic tetraplegia. All participants were implanted in the hand and arm area on the precentral gyrus^87^ of dominant motor cortex with two, 96-channel microelectrode intracortical arrays (1.5 mm electrode length, Blackrock Microsystems, Salt Lake City, UT). Participant T8 was a 53-year-old right-handed male with C4-level AIS-A spinal cord injury that occurred 8 years prior to implant; T5 was a 63-year-old right-handed male with C4-level AIS-C spinal cord injury; and T9 was a 52-year-old right-handed male with tetraplegia due to ALS. Surgical details can be found at^25,30,88^ for each respective participant.

### Neural recordings and feature extraction

In all participants, broad-band neural recordings were sampled at 30 kHz and pre-processed in Simulink using the xPC real-time operating system (The Mathworks Inc., Natick, MA, US; RRID: SCR_014744). From each pre-processed channel, extraction of two neural features from non-overlapping 20 millisecond time bins was performed in real time, as illustrated in Fig. 1A. These included 192 unsorted threshold crossing (TC) and 192 spike band power (SBP) features. Here, we evaluated the neural space by characterizing TC and SBP features as opposed to sorted single units, in order for our results to be directly applicable to iBCI systems, and because iBCI performance has been shown to be comparable when derived from thresholded data as opposed to spike-sorted data^89–92^. Supplementary Figs. S8–S11 illustrate a visual comparison of neural activity from threshold crossings and sorted single units extracted from identical channels. Unless otherwise stated, all offline analyses were performed using MATLAB software (The Mathworks Inc., Natick, MA, US; RRID: SCR_001622). Neural recordings, multiunit feature extraction methods, and single unit sorting methods for this study are described in more detail within the Supplementary Information.

### Behavioral task

During data collection, all participants observed, imagined, and attempted producing three discrete forces (light < medium < hard) with the dominant hand, using either a power or a pincer grasp, over multiple data collection sessions. T8 completed eight sessions between trial days 511–963 relative to the date of his microelectrode array implant surgery; T9 completed one session on trial day 736; and T5 completed two sessions on trial days 365 and 396. Supplementary Table S1 lists all relevant sessions and their associated task parameters.

Each session consisted of multiple 4-minute data collection blocks, as illustrated in Fig. 1B. During each block, one volitional state (observe, imagine, or attempt) and one hand grasp type (power or pincer) were presented. Blocks were arranged in a pseudorandom order, in which volitional states were assigned randomly to each set of three blocks chronologically. This ensured an approximately equal number of blocks per volitional state, distributed evenly across the entire session. All blocks consisted of approximately 20 trials. Participants were encouraged to take an optional break between experimental blocks.

During each trial, participants were prompted to use kinesthetic imagery^93,94^ to internally emulate a) a “light” squeezing force, b) “medium” force, c) “hard” force, d) a finger wiggling movement, or e) rest, with the dominant hand. Finger wiggling trials served as a kinematic control for the force trials. Trials were presented in a pseudorandom order by repeatedly cycling through a complete, randomized set of five trial types until the end of the block.

Participants received audio cues indicating which force to produce (prep phase), when to produce it (go phase), and when to relax (stop phase). Participants also observed a researcher squeezing one of six graspable objects corresponding to light, medium, and hard forces (no object was squeezed during “rest” trials). These objects were grouped into two sets of three, corresponding to forces embodied using a power grasp (sponge = light, stress ball = medium, tennis ball = hard) and a pincer grasp (cotton ball = light, nasal aspirator tip = medium, eraser = hard), as shown in Fig. 1B. At the start of each trial, the researcher presented an object indicating the force level to be observed, imagined, or attempted. At the end of the prep phase (at the beginning of the go phase), the researcher squeezed the object. The prep phase lasted between 2.7 and 3.3 seconds. The variability in the prep phase time reduced confounding effects from anticipatory activity. The researcher squeezed the object during the go phase (3–5 seconds), and then released the object at the beginning of the stop phase (3–5 seconds). These visual cues were presented during the majority of experimental trials in a third-person, frontal perspective, in which the researcher faced the participants while squeezing the objects. Visual cues were presented during the majority of force trials; however, during two experimental sessions, visual cues were omitted during some trials to determine whether force-related information resulted from the presence of objects. Supplementary Figs. S1 and S2 visually compare neural feature activity recorded during these trials with feature activity recorded when both audio and visual cues were presented.

During force observation blocks, participants simply observed these actions without engaging in any activity. During force imagery and attempt blocks, participants imagined generating or attempted to generate sufficient force to “crush” the object squeezed by the researcher. For force imagery blocks, participants were instructed to mentally rehearse the forces needed to crush the various objects, without actually attempting to generate these forces.

To assess the extent to which participants embodied discrete forces during each volitional state, participants completed a Kinesthetic Force Imagery Questionnaire (KFIQ), adapted from the Kinesthetic and Visual Imagery Questionnaire (KVIQ)^81^ after each experimental block. Briefly, participants rated on a scale of 0–10 how vividly they kinesthetically emulated various force levels during light, medium, hard, and wiggling force trials, where 0 = no force embodiment, and 10 = embodiment as intense as able-bodied force execution. The KFIQ is described in more detail within the Supplementary Information.

### Effects of audio vs. audiovisual cues

During all experimental sessions presented in Figs. 2–7, participants received audio cues indicating which forces to produce and when to embody these forces. Participants also received visual cues during most sessions, in which they observed a researcher squeezing objects corresponding to the forces they were asked to observe, imagine, or attempt to produce. These visual cues were presented in order to encourage participants to emulate light, medium, and hard forces within the framework of a real-world setting. The goal was to elicit appropriate neural responses to the force task by providing participants with meaningful visual information about which forces they were emulating. More specifically, the visual cues were implemented to make the physical concept of light, medium, and hard forces seem less abstract to the participants after several years of deafferentation.

To determine the extent of any resulting confounds of object size, hand grasp shape, and other factors within the neuronal responses to the force task, additional supplemental data was collected from participant T8, who was cued to observe, imagine, or attempt producing forces using either only audio cues or both audio and visual cues. These additional data, which are summarized in Supplementary Table S2, are solely presented within the Supplementary Information and do not appear in the main text. Within each supplemental session, correlation coefficients were computed between pairs of trial-averaged feature time courses that resulted from audio-only cues (a) and audiovisual cues (av). These correlations were computed within each volitional state, and within each phase of the task (prep, go, stop), in order to discern whether neuronal responses to the visual cues varied by volitional state or trial phase. For each individual session, this correlational analysis was performed on 120 neural features with the highest signal to noise ratio. Additional methods can be found in the Supplementary Materials.

### Assessment of kinetic vs. kinematic activity

Prior to determining the effects of volitional state on neural force representation in the main dataset, we performed an initial analysis to determine whether force-related modulation of neural feature activity was distinct from modulation to kinematic activity. Briefly, correlation coefficients were computed between pairs of trial-averaged go-phase feature modulation time courses during force (light, medium, hard) and finger-wiggling trials, within each volitional state. For each participant, this analysis was performed on 120 neural features with the highest signal to noise ratio (SNR), as described and presented in the Supplementary Information.

### Characterization of individual features

The first goal of this study was to characterize the tuning properties of individual features. Neural activity resulting from the three *volitional states* (observe, imagine, attempt) and the three discrete *forces* (light, medium, hard) resulted in nine conditions of interest. For each condition, each neural feature’s peristimulus time histogram (PSTH) was computed by averaging the neural activity over go-phase-aligned trials, which were temporally smoothed with a Gaussian kernel (100-ms standard deviation) to aid in visualization.

Additionally, the effect of discrete *force* levels and *volitional states* on the activity of individual features was assessed. This analysis consisted of feature pre-processing performed in MATLAB, as well as statistical analysis implemented within the WRS2 library in the R programming language^95^. Features were pre-processed to calculate each neural feature’s mean deviation from baseline during the go phase of each trial. For each feature, baseline activity was computed by averaging neural activity across multiple “rest” trials. Next, trial-averaged baseline activity was subtracted from neural activity that occurred during individual experimental trials. Finally, the resulting activity traces were averaged across multiple time points spanning each trial’s go phase, which yielded a collection of go-phase neural deviations from baseline.

In R, it was determined that the distribution of go-phase neural deviations passed normality tests (analysis of Q-Q plots and Shapiro-Wilk test, p < 0.05) but was heteroskedastic (Levene’s test, p < 0.05). Thus, a robust 2-way Welch ANOVA on the untrimmed means was implemented to evaluate the main effects (force and VoS), as well as their interaction (p < 0.05). Features that demonstrated a significant interaction between force and VoS were further separated into individual VoS conditions (observe, imagine, attempt). Within each VoS condition, a 1-way Welch ANOVA was implemented on go-phase neural deviations to find features that had significant force tuning (p < 0.05). All p-values were corrected for multiple comparisons using the Benjamini–Hochberg procedure^96^. Taken together, the results of the 1-way and 2-way Welch ANOVA constituted the tuning properties for each individual feature during a given experimental session.

### Neural population analysis and decoding

In addition to characterizing individual features, this work sought to elucidate how much volitional state affects the neural representation of force at the population level. To this end, similarity analysis was used to examine the intrinsic relationship between the activity patterns observed across conditions^51,97,98^. This approach is based on pair-wise comparisons between neural activity patterns and makes no *a priori* assumptions about the structure of the data. T-distributed Stochastic Neighbor Embedding (t-SNE)^52^ was used to project these pair-wise measurements into a low-dimensional representation, which facilitated statistical analysis as well as data visualization while still capturing the relationship between individual trials. This dimensionality reduction technique is well suited to similarity analysis, because it explicitly attempts to preserve nearest-neighbor relationships in the data by minimizing KL-divergence between local neighborhood probability functions in the high and low dimensional spaces. That is, it explicitly attempts to preserve relationships between data points that are close together in the high-dimensional space, which makes it ideal for analyzing neural datasets that lie on or close to a nonlinear manifold. In the low-dimensional “similarity space”, a neural activity pattern is represented by a single point. The distance between points denotes the degree of similarity between the activity patterns they represent. Two identical activity patterns correspond to the same point in this space; the more different they are, the farther apart they lie in the similarity space projection.

We have previously used this method to compare spike trains using point process distance metrics^51^. In the present study, the algorithm was adjusted to operate on continuous (binned) data, in order to include threshold crossing (TC) and spike band power (SPB) extracted features. This modified technique is referred to as continuous similarity (CSIM) analysis. Similarity between feature vectors was evaluated as one minus the cosine of the angle between them. Note that this metric is not affected by the magnitude of the vectors; this property made the analysis more robust to non-stationarities in the data. All features were binned using non-overlapping 20-ms windows, and then smoothed with a 20-bin (400 ms) Gaussian kernel. Two-dimensional CSIM projections were derived over the entire duration of the go-phase. After CSIM was applied, all trials were labeled *post-hoc* with their associated forces and volitional states, in order to visualize how these experimental parameters affected the degree of similarity between neural activity patterns. Here, the goal was to visualize how force and volitional state affected the activity of the neural population, as represented by the low-dimensional CSIM manifold.

The force- and VoS-related information content within this manifold was quantified in two ways. First, force- and VoS-related clustering of trials was evaluated by comparing the distributions of CSIM distances within and between conditions using a Kruskal-Wallis test. The neural space was identified as representing volitional state when the median CSIM distances between trials with the same volitional states were smaller than the distances between trials with different volitional states. Similarly, when the median CSIM distances between trials with the same force were smaller than the distances between trials with different forces, the neural space was identified as representing force.

Second, CSIM was also used to generate discrete state force and volitional state classifiers. TC and SBP features were projected onto a 10 dimensional CSIM representation using data from a 400 ms sliding window, stepped in 100 ms increments. For each window, an LDA classifier was used to generate time-dependent force and volitional state decoding accuracies, using 10-fold cross-validation. Additionally, the effect of the analysis window size on classification accuracy was examined, starting with 100 ms following the go cue and increasing window length in 100 ms increments up to three seconds. For both sets of analyses, force decoding accuracies were determined within each volitional state, in order to determine how volitional state affected the degree to which force-related information was represented within the neural space. These decoding accuracies were compared to the empirical chance distribution of decoding accuracies, estimated using 10,000 random shuffles of trial labels.

## Supplementary information

Supplementary Information.

## Data Availability

The data can be made available upon reasonable request by contacting the lead or senior authors.

## Code availability

Source code for the CSIM algorithm is publicly available at https://donoghuelab.github.io/SSIMS-Analysis-Toolbox/.

## References

[1] Carmena JM (2003). Learning to control a brain-machine interface for reaching and grasping by primates. PLoS Biol..

[2] Evarts EV (1968). Relation of pyramidal tract activity to force exerted during voluntary movement. J. Neurophysiol..

[3] Evarts EV, Fromm C, Kroller J, Jennings VA (1983). Motor Cortex control of finely graded forces. J. Neurophysiol..

[4] Fetz EE, Cheney PD (1980). Postspike facilitation of forelimb muscle activity by primate corticomotoneuronal cells. J. Neurophysiol..

[5] Georgopoulos AP, Kalaska JF, Caminiti R, Massey JT (1982). On the relations between the direction of two-dimensional arm movements and cell discharge in primate motor cortex. J. Neurosci..

[6] Georgopoulos AP, Schwartz AB, Kettner RE (1986). Neuronal population coding of movement direction. Sci..

[7] Humphrey DR (1970). A chronically implantable multiple micro-electrode system with independent control of electrode positions. Electroencephalogr. Clin. Neurophysiol..

[8] Kakei S, Hoffman DS, Strick PL (1999). Muscle and movement representations in the primary motor cortex. Sci..

[9] Morrow MM, Miller LE (2003). Prediction of muscle activity by populations of sequentially recorded primary motor cortex neurons. J. Neurophysiol..

[10] Oby, E. R. *et al*. In *Statistical Signal Processing for Neuroscience and Neurotechnology* 369-406 (Elsevier Inc., 2010).

[11] Pohlmeyer EA, Solla SA, Perreault EJ, Miller LE (2007). Prediction of upper limb muscle activity from motor cortical discharge during reaching. J. Neural Eng..

[12] Sergio LE, Kalaska JF (2003). Systematic changes in motor cortex cell activity with arm posture during directional isometric force generation. J. Neurophysiol..

[13] Vargas-Irwin CE (2010). Decoding complete reach and grasp actions from local primary motor cortex populations. J. Neurosci..

[14] Zhuang J, Truccolo W, Vargas-Irwin C, Donoghue JP (2010). Decoding 3-D reach and grasp kinematics from high-frequency local field potentials in primate primary motor cortex. IEEE Trans. Biomed. Eng..

[15] Hermes D (2011). Functional MRI-based identification of brain areas involved in motor imagery for implantable brain-computer interfaces. J. Neural Eng..

[16] Hochberg LR (2006). Neuronal ensemble control of prosthetic devices by a human with tetraplegia. Nat..

[17] Jarosiewicz B (2015). Virtual typing by people with tetraplegia using a self-calibrating intracortical brain-computer interface. Sci. Transl. Med..

[18] Kim SP, Simeral JD, Hochberg LR, Donoghue JP, Black MJ (2008). Neural control of computer cursor velocity by decoding motor cortical spiking activity in humans with tetraplegia. J. Neural Eng..

[19] Kim SP (2011). Point-and-click cursor control with an intracortical neural interface system by humans with tetraplegia. IEEE Trans. Neural Syst. Rehabil. Eng..

[20] Kubler A (2005). Patients with ALS can use sensorimotor rhythms to operate a brain-computer interface. Neurol..

[21] Leuthardt EC, Schalk G, Wolpaw JR, Ojemann JG, Moran DW (2004). A brain-computer interface using electrocorticographic signals in humans. J. Neural Eng..

[22] Schalk G (2008). Two-dimensional movement control using electrocorticographic signals in humans. J. Neural Eng..

[23] Simeral JD, Kim SP, Black MJ, Donoghue JP, Hochberg LR (2011). Neural control of cursor trajectory and click by a human with tetraplegia 1000 days after implant of an intracortical microelectrode array. J. Neural Eng..

[24] Wolpaw JR, Birbaumer N, McFarland DJ, Pfurtscheller G, Vaughan TM (2002). Brain-computer interfaces for communication and control. Clin. Neurophysiol..

[25] Gilja V (2015). Clinical translation of a high-performance neural prosthesis. Nat. Med..

[26] Pandarinath, C. *et al*. High performance communication by people with paralysis using an intracortical brain-computer interface. *Elife*, **6**, 10.7554/eLife.18554 (2017). 10.7554/eLife.18554 PMC5319839 28220753

[27] Collinger JL (2013). High-performance neuroprosthetic control by an individual with tetraplegia. Lancet.

[28] Hochberg LR (2012). Reach and grasp by people with tetraplegia using a neurally controlled robotic arm. Nat..

[29] Wodlinger B (2015). Ten-dimensional anthropomorphic arm control in a human brain-machine interface: difficulties, solutions, and limitations. J. Neural Eng..

[30] Ajiboye A Bolu, Willett Francis R, Young Daniel R, Memberg William D, Murphy Brian A, Miller Jonathan P, Walter Benjamin L, Sweet Jennifer A, Hoyen Harry A, Keith Michael W, Peckham P Hunter, Simeral John D, Donoghue John P, Hochberg Leigh R, Kirsch Robert F (2017). Restoration of reaching and grasping movements through brain-controlled muscle stimulation in a person with tetraplegia: a proof-of-concept demonstration. The Lancet.

[31] Bouton CE (2016). Restoring cortical control of functional movement in a human with quadriplegia. Nat..

[32] Ethier C, Oby ER, Bauman MJ, Miller LE (2012). Restoration of grasp following paralysis through brain-controlled stimulation of muscles. Nat..

[33] Moritz CT, Perlmutter SI, Fetz EE (2008). Direct control of paralysed muscles by cortical neurons. Nat..

[34] Pohlmeyer EA (2009). Toward the restoration of hand use to a paralyzed monkey: brain-controlled functional electrical stimulation of forearm muscles. PLoS One.

[35] Flint RD (2014). Extracting kinetic information from human motor cortical signals. Neuroimage.

[36] Downey JE (2018). Implicit Grasp Force Representation in Human Motor Cortical Recordings. Front. Neurosci..

[37] Hatsopoulos NG, Suminski AJ (2011). Sensing with the motor cortex. Neuron.

[38] Sanes JN, Donoghue JP (2000). Plasticity and primary motor cortex. Annu. Rev. Neurosci..

[39] Kalaska JF (2009). From intention to action: motor cortex and the control of reaching movements. Adv. Exp. Med. Biol..

[40] Filimon F, Nelson JD, Hagler DJ, Sereno MI (2007). Human cortical representations for reaching: mirror neurons for execution, observation, and imagery. Neuroimage.

[41] Grezes J, Decety J (2001). Functional anatomy of execution, mental simulation, observation, and verb generation of actions: a meta-analysis. Hum. Brain Mapp..

[42] Miller KJ (2010). Cortical activity during motor execution, motor imagery, and imagery-based online feedback. Proc. Natl Acad. Sci. USA.

[43] Porro CA (1996). Primary motor and sensory cortex activation during motor performance and motor imagery: a functional magnetic resonance imaging study. J. Neurosci..

[44] Vargas-Irwin, C. E. *et al*. Watch, Imagine, Attempt: Motor Cortex Single-Unit Activity Reveals Context-Dependent Movement Encoding in Humans With Tetraplegia. *Frontiers in Human N**euroscience*, **12**, 10.3389/fnhum.2018.00450 (2018). 10.3389/fnhum.2018.00450 PMC6262367 30524258

[45] Jeannerod M (2001). Neural simulation of action: a unifying mechanism for motor cognition. Neuroimage.

[46] Mukamel R, Ekstrom AD, Kaplan J, Iacoboni M, Fried I (2010). Single-neuron responses in humans during execution and observation of actions. Curr. Biol..

[47] Page SJ, Levine P, Leonard A (2007). Mental practice in chronic stroke: results of a randomized, placebo-controlled trial. Stroke.

[48] Page SJ, Levine P, Sisto S, Johnston MV (2001). A randomized efficacy and feasibility study of imagery in acute stroke. Clin. Rehabil..

[49] Murphy BA, Miller JP, Gunalan K, Ajiboye AB (2016). Contributions of Subsurface Cortical Modulations to Discrimination of Executed and Imagined Grasp Forces through Stereoelectroencephalography. PLoS One.

[50] Cramer SC, Lastra L, Lacourse MG, Cohen MJ (2005). Brain motor system function after chronic, complete spinal cord injury. Brain.

[51] Vargas-Irwin CE, Brandman DM, Zimmermann JB, Donoghue JP, Black MJ (2015). Spike train SIMilarity Space (SSIMS): a framework for single neuron and ensemble data analysis. Neural Comput..

[52] van der Maaten L, Hinton G (2008). Visualizing Data using t-SNE. J. Mach. Learn. Res..

[53] Kalaska JF, Cohen DA, Hyde ML, Prud’homme M (1989). A comparison of movement direction-related versus load direction-related activity in primate motor cortex, using a two-dimensional reaching task. J. Neurosci..

[54] Keisker B, Hepp-Reymond MC, Blickenstorfer A, Meyer M, Kollias SS (2009). Differential force scaling of fine-graded power grip force in the sensorimotor network. Hum. Brain Mapp..

[55] Neely KA, Coombes SA, Planetta PJ, Vaillancourt DE (2013). Segregated and overlapping neural circuits exist for the production of static and dynamic precision grip force. Hum. Brain Mapp..

[56] Rearick MP, Johnston JA, Slobounov SM (2001). Feedback-dependent modulation of isometric force control: an EEG study in visuomotor integration. Brain Res. Cogn. Brain Res.

[57] Wang K (2017). A brain-computer interface driven by imagining different force loads on a single hand: an online feasibility study. J. Neuroeng. Rehabil..

[58] Dushanova J, Donoghue J (2010). Neurons in primary motor cortex engaged during action observation. Eur. J. Neurosci..

[59] Kraskov A (2014). Corticospinal mirror neurons. Philos. Trans. R. Soc. Lond. B Biol. Sci..

[60] Mazurek KA, Rouse AG, Schieber MH (2018). Mirror Neuron Populations Represent Sequences of Behavioral Epochs During Both Execution and Observation. J. Neurosci..

[61] Vigneswaran G, Philipp R, Lemon RN, Kraskov A (2013). M1 corticospinal mirror neurons and their role in movement suppression during action observation. Curr. Biol..

[62] Lanzilotto M (2019). Anterior Intraparietal Area: A Hub in the Observed Manipulative Action Network. Cereb. Cortex.

[63] Caggiano V, Fogassi L, Rizzolatti G, Thier P, Casile A (2009). Mirror neurons differentially encode the peripersonal and extrapersonal space of monkeys. Sci..

[64] Umilta MA (2001). I know what you are doing. a neurophysiological study. Neuron.

[65] Caggiano V (2011). View-based encoding of actions in mirror neurons of area f5 in macaque premotor cortex. Curr. Biol..

[66] Maranesi M, Livi A, Bonini L (2017). Spatial and viewpoint selectivity for others’ observed actions in monkey ventral premotor mirror neurons. Sci. Rep..

[67] Alaerts K, de Beukelaar TT, Swinnen SP, Wenderoth N (2012). Observing how others lift light or heavy objects: time-dependent encoding of grip force in the primary motor cortex. Psychol. Res..

[68] Green JB, Sora E, Bialy Y, Ricamato A, Thatcher RW (1999). Cortical motor reorganization after paraplegia: an EEG study. Neurol..

[69] Lacourse MG, Cohen MJ, Lawrence KE, Romero DH (1999). Cortical potentials during imagined movements in individuals with chronic spinal cord injuries. Behav. Brain Res..

[70] Ghez C, Gordon J, Ghilardi MF (1995). Impairments of reaching movements in patients without proprioception. II. Effects of visual information on accuracy. J. Neurophysiol..

[71] Gordon J, Ghilardi MF, Ghez C (1995). Impairments of reaching movements in patients without proprioception. I. Spatial errors. J. Neurophysiol..

[72] Sainburg RL, Poizner H, Ghez C (1993). Loss of proprioception produces deficits in interjoint coordination. J. Neurophysiol..

[73] Tan DW (2014). A neural interface provides long-term stable natural touch perception. Sci. Transl. Med..

[74] Ramos-Murguialday A (2012). Proprioceptive feedback and brain computer interface (BCI) based neuroprostheses. PLoS One.

[75] Shaikhouni A, Donoghue JP, Hochberg LR (2013). Somatosensory responses in a human motor cortex. J. Neurophysiol..

[76] Schiefer MA, Graczyk EL, Sidik SM, Tan DW, Tyler DJ (2018). Artificial tactile and proprioceptive feedback improves performance and confidence on object identification tasks. PLoS One.

[77] Tabot GA, Kim SS, Winberry JE, Bensmaia SJ (2015). Restoring tactile and proprioceptive sensation through a brain interface. Neurobiol. Dis..

[78] Brochier T, Boudreau MJ, Pare M, Smith AM (1999). The effects of muscimol inactivation of small regions of motor and somatosensory cortex on independent finger movements and force control in the precision grip. Exp. Brain Res..

[79] Carteron A (2016). Temporary Nerve Block at Selected Digits Revealed Hand Motor Deficits in Grasping Tasks. Front. Hum. Neurosci..

[80] Solstrand Dahlberg L, Becerra L, Borsook D, Linnman C (2018). Brain changes after spinal cord injury, a quantitative meta-analysis and review. Neurosci. Biobehav. Rev..

[81] Malouin F (2007). The Kinesthetic and Visual Imagery Questionnaire (KVIQ) for assessing motor imagery in persons with physical disabilities: a reliability and construct validity study. J. Neurol. Phys. Ther..

[82] Fabbri-Destro M, Rizzolatti G (2008). Mirror neurons and mirror systems in monkeys and humans. Physiol..

[83] Chase SM, Schwartz AB, Kass RE (2009). Bias, optimal linear estimation, and the differences between open-loop simulation and closed-loop performance of spiking-based brain-computer interface algorithms. Neural Netw..

[84] Koyama S (2010). Comparison of brain-computer interface decoding algorithms in open-loop and closed-loop control. J. Comput. Neurosci..

[85] Jarosiewicz B (2013). Advantages of closed-loop calibration in intracortical brain-computer interfaces for people with tetraplegia. J. Neural Eng..

[86] Flesher SN (2016). Intracortical microstimulation of human somatosensory cortex. Sci. Transl. Med..

[87] Yousry TA (1997). Localization of the motor hand area to a knob on the precentral gyrus. A new landmark. Brain.

[88] Nuyujukian P (2018). Cortical control of a tablet computer by people with paralysis. PLoS One.

[89] Chestek CA (2011). Long-term stability of neural prosthetic control signals from silicon cortical arrays in rhesus macaque motor cortex. J. Neural Eng..

[90] Christie BP (2015). Comparison of spike sorting and thresholding of voltage waveforms for intracortical brain-machine interface performance. J. Neural Eng..

[91] Fraser GW, Chase SM, Whitford A, Schwartz AB (2009). Control of a brain-computer interface without spike sorting. J. Neural Eng..

[92] Trautmann EM (2019). Accurate Estimation of Neural Population Dynamics without Spike Sorting. Neuron.

[93] Mizuguchi N, Nakamura M, Kanosue K (2017). Task-dependent engagements of the primary visual cortex during kinesthetic and visual motor imagery. Neurosci. Lett..

[94] Stevens JA (2005). Interference effects demonstrate distinct roles for visual and motor imagery during the mental representation of human action. Cognition.

[95] Wilcox, R. R. *Introdction to Robust Estimation and Hypothesis Testing*. 3 edn, (Academic Press, 2017).

[96] Benjamini Y, Hochberg Y (1995). Controlling the False Discovery Rate - a Practical and Powerful Approach to Multiple Testing. J. R. Stat. Soc. B.

[97] Kriegeskorte N, Mur M, Bandettini P (2008). Representational similarity analysis - connecting the branches of systems neuroscience. Front. Syst. Neurosci..

[98] Shepard RN, Chipman S (1970). Second-Order Isomorphism of Internal Representations - Shapes of States. Cognit. Psychol..

