# Supplementary material for: Neural Representation of Observed, Imagined, and Attempted Grasping Force in Motor Cortex of Individuals with Chronic Tetraplegia
Source: Sci Rep. 2020 Jan 29;10:1429. doi: 10.1038/s41598-020-58097-1 (PMC6989675; doi:10.1038/s41598-020-58097-1)
Supplement: Supplementary file 1 — Supplementary Information. [file 41598_2020_58097_MOESM1_ESM.docx]

**Supplementary Information for “Neural Representation of Observed, Imagined, and Attempted Grasping Force in Motor Cortex of Individuals with Chronic Tetraplegia”**

**Anisha Rastogi^1^, Carlos Vargas-Irwin^2,3^, Francis Willett^5,6,7^, Jessica Abreu^1,12^, Douglas C. Crowder^1, 12^, Brian Murphy^1, 12^, William Memberg^1^, Jonathan Miller^12,13,15^, Jennifer Sweet^12,13^, Benjamin Walter^12,14^, Sydney Cash^16,19^, Paymon Rezaii^5^, Brian Franco^16^, Jad Saab^3,4,18^, Sergey Stavisky^5,6,7^, Krishna Shenoy^6,7,8,9,10^, Jaimie Henderson^5,7,11^, Leigh Hochberg^4,17,18,19^, Robert Kirsch^1,12^, A. Bolu Ajiboye^1,12*^**

**Supplementary Methods**

**Pre-processing of Neural Data:** In all participants, each intracortical microelectrode array was attached to a percutaneous pedestal connector on the head. Patient cables connected the pedestals to amplifiers (Blackrock Microsystems, Salt Lake City, UT) that bandpass filtered (0.3 Hz – 7.5 kHz) and digitized (30 kHz) the neural signals from each channel on the microelectrode array. These digitized signals were pre-processed in Simulink using the xPC real-time operating system (The Mathworks Inc., Natick, MA, US; RRID: SCR_014744). Each channel was bandpass filtered (250-5000 Hz), common average referenced (CAR), and down-sampled to 15 kHz in real time. CAR was implemented by selecting 60 channels from each microelectrode array that exhibited the lowest variance, and subsequently averaging these channels to yield an array-specific common average reference. This reference signal was subtracted from all individual channels on each of the arrays.

**Extraction of Neural Features:** From each filtered, referenced channel, two multiunit neural features were extracted in real time. These included unsorted threshold crossing (TC) and spike band power (SBP) features, from non-overlapping 20 millisecond time bins, as illustrated in Figure 1A. TC features were defined as the number of times the voltage on each channel crossed a predefined noise threshold within each time bin (-4.5 x RMS for T8; -4.5 x RMS for T5; -3.5 x RMS for T9). These thresholds were hand selected to accept the most action potential signals and to reject the most non-neural noise (Christie et al, 2015; Fraser et al, 2009). RMS was calculated from one minute of neural data recorded at the beginning of each experimental session. SBP features were defined as the root mean square of the signal in the spike band (250-5000 Hz) of each channel, within each time bin. Prior to offline analysis, these features were normalized by subtracting the mean of each feature within each recording block, in order to minimize non-stationarities in the data.

**Extraction of Sorted Single Units:** In addition to the multiunit features extracted and analyzed in the main text, sorted single units were extracted offline from each channel of neural activity for participant T8. Spike sorting was performed using the Wave_clus algorithm in Matlab (Quiroga et al., 2004). Here, noise thresholds for single unit spike detection were equivalent to those implemented for TC multiunit features on corresponding recording channels. Next, the effect of discrete *force* levels and *volitional states* on the activity of single units was assessed by performing robust 2-way Welch ANOVA analysis on single unit activity. This analysis was implemented as described in the main text Methods section.

**Kinesthetic Force Imagery Questionnaire (KFIQ):** Participants completed the KFIQ, adapted from the Kinesthetic and Visual Imagery Questionnaire (KVIQ) (Malouin et al, 2007) after each experimental block. The KFIQ was implemented to assess how intensely participants embodied the act of producing grasping forces during each volitional state, and during each force level. (During Session 12, participant T5 completed the KFIQ once at the end of the session rather than after every block, in order to minimize cognitive fatigue.) For each of the three volitional states, participants rated on a scale of 0-10 how vividly they were able to kinesthetically emulate various force levels, where a score of zero indicated no embodiment, and a score of ten indicated embodiment as intense as able-bodied execution of the force. Participants were specifically instructed to rate how intensely they *felt* themselves generating forces with the dominant hand, rather than rating how intensely they visualized producing them, in order to best capture the kinesthetic aspect of the task. KFIQ scores during the observed, imagined, and attempted force trials were then correlated with neural activity during the three volitional states. Additionally, participants were asked to rate the degree to which they kinesthetically emulated force production during finger wiggling trials, which, in theory, required minimal kinetic and maximal kinematic output.

**Assessment of Neural Activity due to Audio vs. Audiovisual Cues:** As described in the main text, force trials were prompted with both audio and visual cues during most sessions. Specifically, a researcher squeezed one of six graspable objects corresponding to light, medium, and hard forces produced via power and pincer grasping. These visual cues, which gave participants real-world context of the forces they were instructed to emulate, nonetheless posed a risk of introducing extraneous activity in the neural force data due to hand aperture and the presence of graspable objects of various sizes and shapes. Therefore, five supplementary datasets, plus on dataset from the main text, were analyzed to determine the extent to which neural modulation was influenced by the presence or absence of visual cues. During these sessions, participants observed, imagined, or attempted forces with and without visual cues (see Supplementary Table S2). Trials prompted with both audio and visual cues are referred to as audiovisual (av) trials, while audio-only (a) trials were prompted solely with audio cues.

In this analysis, correlations were computed between neural features during a vs. av trials. Here, the analysis was performed on 120 features with the highest signal to noise ratio (SNR), which were isolated via the following pre-processing steps. Neural activity resulting from the three *volitional states* (observe, imagine, attempt) and the three discrete *forces* (light, medium, hard) resulted in nine conditions of interest. For each condition, each neural feature’s SNR was computed by averaging the neural activity over go-phase-aligned trials, dividing this mean activity by the standard deviation of the go-phase-aligned activity, and subsequently averaging this result over all time points of the trial. This resulted in nine SNR values per feature, which were averaged to obtain one SNR value per feature spanning all forces and volitional states.

For each of the 120 features with highest SNR, correlation coefficients were computed between pairs of trial-averaged PSTHs during audiovisual (av) and audio-only (a) trials, within each volitional state (observe, imagine, attempt) and trial phase (prep, go, stop). Multi-session distributions of feature correlation coefficients between a-a, av-av, and a-av trial were generated for each volitional state and trial phase. Finally, Kruskal-Wallis tests were implemented on each set of correlation coefficient distributions for each volitional state and trial phase, the Tukey Method was used to correct for multiple comparisons, and the resulting p values were further corrected using the Benjamini-Hochberg method (Benjamini and Hochberg 1995) across all three volitional states and all three trial phases. It was expected that no statistically significant differences would exist between go-phase a-av versus av-av or a-a distributions, which would suggest that go-phase neural activity was not affected by the presence of visual cues.

**Assessment of Kinetic vs. Kinematic Activity:** Prior to determining how force and volitional state modulated neural activity, an initial analysis was performed to determine whether neural modulation to force was distinct from modulation to kinematic activity. In order to visualize the extent to which force and wiggle trials were correlated across volitional states, heat maps of the absolute values of correlation coefficients averaged over 120 features were computed. (The 120 features were chosen to have the highest SNR across forces and volitional states, as described in the “Assessment of Neural Activity due to Audio vs. Audiovisual Cues” section of the Supplementary Information.) Distributions of feature correlation coefficients between force-force and force-wiggle trials were also plotted for each volitional state, to further visualize the extent to which these distributions differed from one another. To quantify the degree of correlation between force and wiggle trials, t-tests were implemented on all pairs of correlation coefficient distributions within each volitional state, as well as across volitional states, and were then corrected using the Benjamini-Hochberg method (Benjamini and Hochberg 1995) across 15 pairs and three volitional states. It was expected that correlations between pairs of force trials would be significantly different from correlations between force and wiggle trials (p<0.05), indicating that neural activity generated during force trials was distinct from activity during kinematic wiggling.

**Supplementary Results**

Description of Experimental Sessions and Audiovisual Cues

**Description of Experimental Sessions in Main Text**

Supplementary Table S1 displays session information for each participant. T8 completed four sessions between trial days 511-536 relative to the date of his implant surgery, in which he emulated discrete forces using a power grasp, and three sessions between trial days 732-963 in which he performed the same force matching task using a pincer grasp. Additionally, T5 completed one session each of power (trial day 365) and pincer (trial day 396) grasping force, and T9 completed one session of pincer grasping forces on trial day 369.

During most sessions, participants received audio and visual cues indicating which force to produce, as described in the main text. However, during some trials of Sessions 2 and 3, visual cues were omitted to determine the extent to which force-related information resulted from the presentation and manipulation of objects by the researcher. During Session 2, the participant solely received audio cues that indicated which force to produce (prep phase), when to produce it (go phase), and when to cease force production (stop phase). During Session 3, the participant received three types of cues during force observation blocks, as indicated in Supplementary Table S1: 1) audio cues only (listen only), 2) audio cues plus the presentation of an object without any subsequent object manipulation (static observe), and 3) audio cues plus the presentation and squeezing of an object, as described in the main text (observe). During *imagine* and *attempt* blocks, participants received both audio and visual cues as indicated in the main text. Here, we treat the *listen only* and *static observe* conditions as two additional volitional states, along with the aforementioned *observe, imagine,* and *attempt* conditions.

Supplementary Figure S1 visually compares the activity of 25 features with the highest SNR from each session in participant T8, during several volitional states. For each session, these 25 features were isolated as described in the Assessment of Kinematic vs. Kinetic Activity portion of the Supplementary Methods and z-scored to aid in feature-to-feature comparisons. Supplementary Figure S1A displays neural activity during Sessions 2 and 3, in which solely audio cues were presented for at least four blocks of experimental trials. In contrast, Supplementary Figures S1B and S1B show neural activity produced during the standard presentation of visual and audio cues described in the main text, during embodied power grasping (Supplementary Figure S1B) and pincer grasping (Supplementary Figure S1C) forces. A visual comparison between Supplementary Figures S1A, S1B, and S1C shows that the introduction of visual cues often resulted in increased neural activity during the preparatory and stop phases of the experimental trial. These peaks in prep- and stop-phase activity were particularly prominent during observed forces. Additionally, prep- and stop-phase activity peaks tended to become less apparent during the progression from passive observation to attempted force production in participant T8. However, go-phase neural activity – during which T8 actively observed, imagined, or attempted force production – remained comparable whether or not the participant received visual cues. We opted present both visual and auditory cues to participants during all trials subsequent to Session 3, in order to encourage cognitive engagement with the force matching task.

| Session No. | Participant, Grasp Type | Post-Implant Day | No. Blocks Per Volitional State | | | | |
| --- | --- | --- | --- | --- | --- | --- | --- |
|  |  |  | **Audio Listen** | **Static Observe** | **Observe** | **Imagine** | **Attempt** |
| 1 | T8, power | 2016.04.25  Day 511 |  | 4 | 4 | 4 | 4 |
| 2 | T8, power | 2016.05.02  Day 518 | 7* |  |  | 7* | 7* |
| 3 | T8, power | 2016.05.09  Day 525 | 4* | 4 | 4 | 4 | 4 |
| 4 | T8, power | 2016.05.20  Day 536 |  |  | 7 | 7 | 7 |
| 5 | T8, power | 2016.06.03  Day 550 |  |  | 7 | 7 | 8 |
| 6 | T8, pincer | 2016.12.02  Day 732 |  |  | 7 | 7 | 7 |
| 7 | T8, pincer | 2016.12.07  Day 737 |  |  | 7 | 7 | 7 |
| 8 | T8, pincer | 2017.07.21  Day 963 |  |  | 5 | 5 | 5 |
| 9 | T9, pincer | 2017.02.15  Day 736 |  |  | 4 | 4 | 4 |
| 10 | T5, power | 2017.08.17  Day 365 |  |  | 4 | 4 | 4 |
| 11 | T5, pincer | 2017.09.17  Day 396 |  |  | 4 | 4 | 4 |

**Supplementary Table S1. Session information.** Session information for participants T8, T9, and T5, including the number of experimental blocks per volitional state. Each block contained approximately 20 force trials (see Figure 1). Visual cues were provided to participants during all sessions except session 2, in which the participant only received audio cues, as indicated by the asterisks. During session 1, T8 completed four blocks in which force-related objects were presented but not squeezed by the researcher (“static observe”), in addition to the usual observe, imagine, and attempt blocks. During session 3, T8 completed four blocks with solely audio cues (“audio listen”); four “static observe” blocks; and four of each of the usual observe, imagine, and attempt blocks. “Audio listen” and “static observe” blocks from Sessions 1 and 3 were excluded from the single-feature and CSIMS analysis presented in the main text.


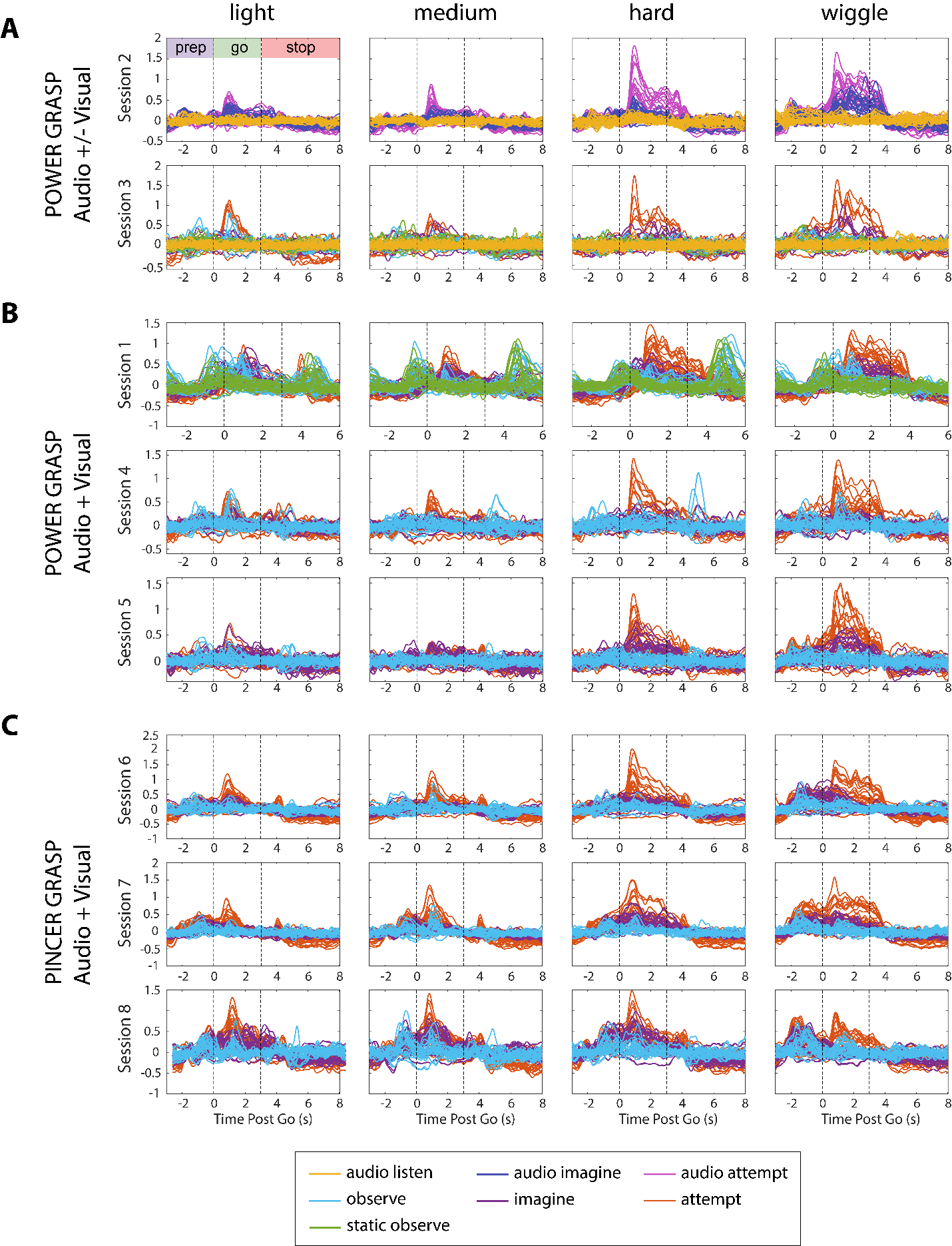


**Supplementary Figure S1. Feature Activity by Volitional State and Cue in Participant T8.** **A.** Go-phase-aligned, normalized feature activity during *audio listen, audio imagine,* and *audio attempt* trials (Session 2) and audio listen*, static observe, observe, imagine,* and *attempt* blocks (Session 3). Note that in Session 2, visual cues were omitted during all trials. **B.** Feature activity during *observed, imagined,* and *attempted* power grasping force production. **C.** Feature activity during *observed, imagined,* and *attempted* pincer grasping force production. During all sessions, the magnitude of neural activity rises during the progression from passive observation to active attempt of forces, regardless of the presence or absence of visual cues. Note the presence of prep- and stop-phase peaks in panels B and C.

**Effects of “Audio Only” vs. “Audiovisual” Cues**

During all experimental sessions presented in the main text, participants received audio cues indicating which force to produce and when to produce it. During a subset of sessions indicated in Supplementary Table S1, participants also received visual cues, in which they observed a researcher squeezing objects corresponding to the forces they were asked to observe, imagine, or attempt, as shown in Figure 1 of the main text.

These visual cues were presented in order to provide concrete, real-world context to the participants about force production, as discussed in the main text. This approach, while valuable from a functional standpoint, has a risk of producing confounds in the neural data that reflect object size, grasp aperture, kinematic neural responses, or additional variables other than force and volitional state. Therefore, prior to implementing visual cues during experimental sessions presented in the main text, supplemental data was collected in order to compare the neural responses to force trials with and without visual in participant T8. These datasets are summarized in Supplementary Table S2.

| Session No. | Post Implant Day | No. Blocks Per Volitional State | | | | | |
| --- | --- | --- | --- | --- | --- | --- | --- |
|  |  | **Audio Listen** | **Audio Imagine** | **Audio Attempt** | **AV**  **Observe** | **AV**  **Imagine** | **AV**  **Attempt** |
| S1 | 2016.02.25  Day 451 |  | 5 |  |  | 2 |  |
| S2 | 2016.03.04  Day 459 |  | 8 |  |  | 3 |  |
| S3 | 2016.04.13  Day 499 |  |  | 3 |  |  | 3 |
| 3 | 2016.05.09  Day 525 | 4 | ` |  | 4 | 4 | 4 |
| S4 | 2016.05.23  Day 539 | 7 |  |  | 7 |  |  |
| S5 | 2016.06.07  Day 554 | 6 |  |  | 6 |  |  |

**Supplementary Table S2. Supplementary Session Information.** Supplementary grasping force session information for participant T8, including the number of experimental blocks per volitional state (observe, imagine, attempt) and cue type (audio only or audiovisual/AV). During audio blocks, visual cues were omitted and only audio cues were used to prompt the participant to observe, imagine, or attempt force production. During audiovisual (AV) blocks, participants received both audio and visual cues, in which a researcher squeezed an object associated with each force level. Note that except for Session 3, all sessions listed in this table are supplementary and do not appear in the main text. All sessions in this table were used to assess the influence of visual cues on observed (Sessions S4, S5), imagined (Sessions S1, S2, S3), and attempted (Session 3) force production.

To elucidate the influence of visual cues on the neural data, correlational analysis was performed on force trials that were prompted with audio cues only (a) and with both audio and visual cues (av). Specifically, each panel of Supplementary Figure S2 shows the mean correlation between pairs of audio-only trials (a-a), pairs of audiovisual trials (av-av), and pairs of audio-only and audiovisual trials (a-av). Here, prep- and stop-phase correlations between a-a, a-av, and av-av trials appear to differ statistically during observed and imagined force production (p<0.05 Tukey method of multiple comparisons, Benjamini Hochberg correction. This suggests that the inclusion of visual cues introduces changes in the neural activity during the preparatory and stop phases of observed and imagined force trials. These changes take the form of prep- and stop-phase peaks in neural activity while visual cues are included, as shown in Supplementary Figure S1.

However, with the exception of imagined force trials, correlations between a-a, a-av, and av-av trials do not differ statistically during the active “go” phase of the experimental trial. These results suggest that the introduction of visual cues do not introduce confounds in go-phase trial activity. Furthermore, Supplementary Figure S3, which illustrates population-level go-phase activity during a session in which visual cues were omitted, shows that the neural activity follows the same trends as presented in the main text. Specifically, volitional state is represented to a greater extent than force, and attempted forces are more discriminable than observed and imagined forces (Figures 4-6). Therefore, while preparatory and stop phase activity was affected, the introduction of visual cues did not seem to influence the go-phase neural activity, from which the major results presented in the main text are derived.


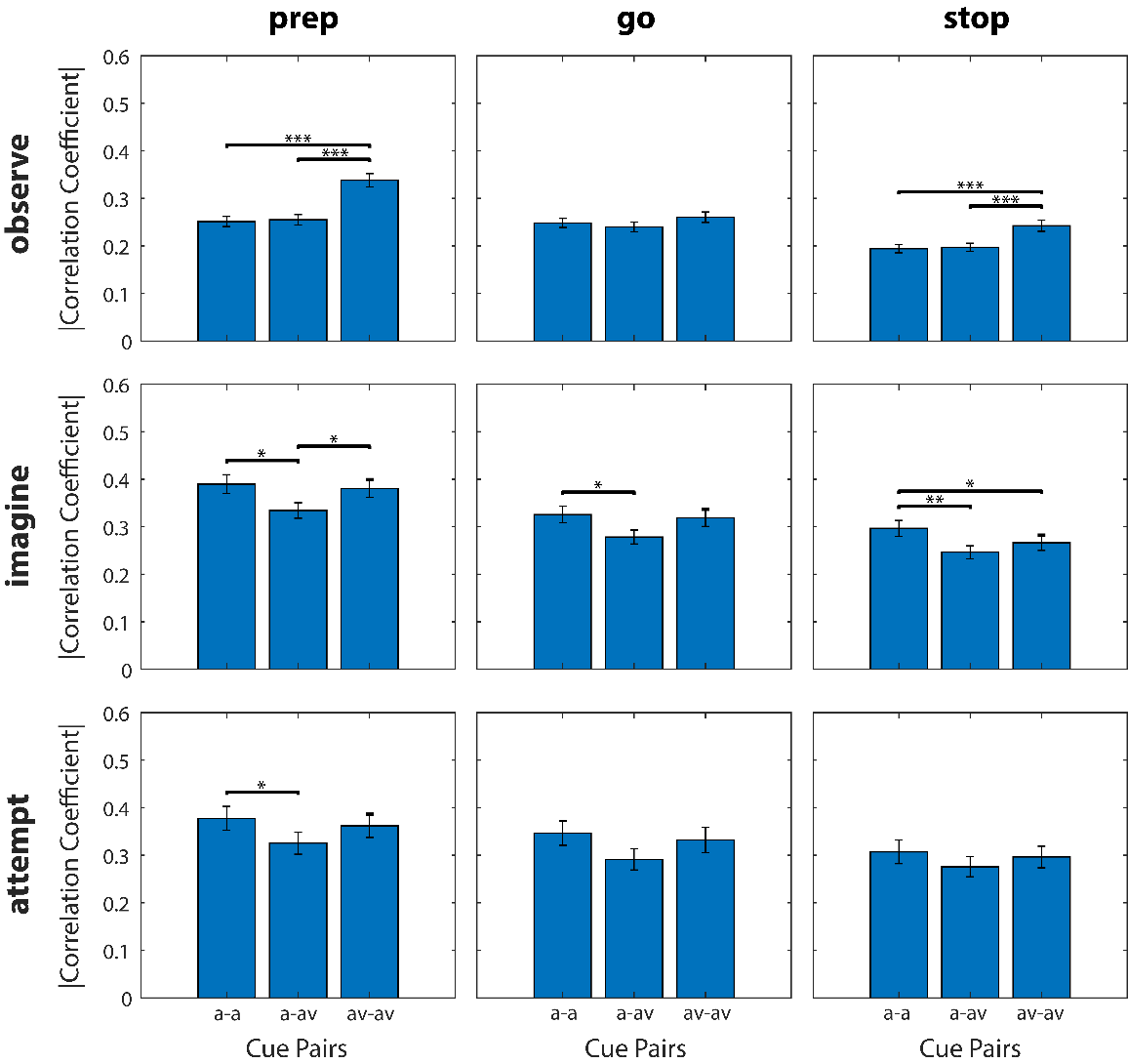


**Supplementary Figure S2. Mean correlations between audio-only (a) and audiovisual (av) trials.**  Mean feature correlations between pairs of a-a, av-av, and a-av trials are depicted for each volitional state (rows) and trial phase (columns), averaged over multiple sessions by volitional state. Asterisks indicate significance at the p<0.05 (*), p<0.01 (**), and p<0.001(***) level. P values were adjusted for multiple comparisons (Tukey Method) and corrected across volitional states and trial phases (Benjamini Hochberg method). Error bars indicate 95% confidence intervals.

In addition to the results of this correlational analysis, Supplementary Figure S3 exhibits population-level neural activity during Session 2, during which visual cues were entirely omitted from the behavioral task. Supplementary Figure S3A exhibits clustering of experimental trials by volitional state, as well as force-related clustering that is most evident during attempted force production. Furthermore, as indicated by the CSIM space classification accuracies for volitional state (76%), observed force (33%), imagined force (55%), and attempted force (67%), volitional state representation in the neural space is stronger than force representation. Finally, as predicted by the results in the main text, attempted forces are more discriminable than observed and imagined forces. Supplementary Figure S3B further illustrates the representation of individual volitional states. Critically, the trends exhibited within this supplementary figure are nearly identical to the findings outlined in the main text. Therefore, taken together, Supplementary Figures S2 and S3 suggest that the introduction of visual cues do not influence the major trends identified in the main study.


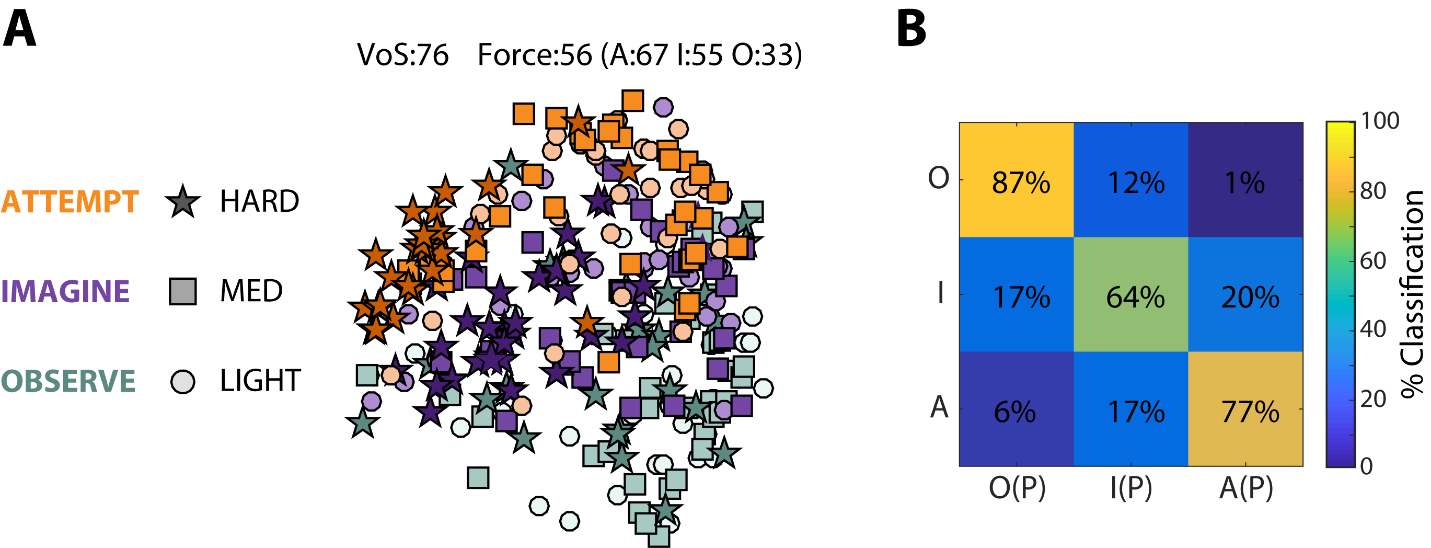


**Supplementary Figure S3. CSIM Neural Population Data for Session 2 (participant T8).**  **A.** Two-dimensional CSIM plot for Session 2. Each point represents the activity of the entire population of simultaneously-extracted features during a single trial. The distance between points indicates the degree of similarity between trials. Clustering of similar symbols denotes similarity between trials with the same intended force level, while clustering of similar colors denotes similarity between trials with the same volitional state. LDA classification accuracies for volitional state and observed (O), imagined (I), and attempted (A) forces from the CSIM space are indicated in the panel title. **B.** Feature ensemble volitional state confusion matrix for Session 2. Offline decoding accuracies were computed using an LDA classifier implemented within a 10-dimensional CSIM representation of the neural data, using 10-fold cross-validation, over a 400-ms sliding window stepped down in 100 ms increments Classification accuracies for individual volitional states were averaged over all time points within the go phase of the behavior task. Note that the mean of the empirical chance distribution (averaged across participants) was 33%, with 95% of samples between 26.6 and 40.3%.

Characterization of Individual Features

**Assessment of Kinetic vs. Kinematic Activity**

To determine whether neural modulation to force was distinct from modulation to kinematic activity, an initial correlational analysis was performed between neural data collected during force trials and neural data collected during finger wiggling trials. Specifically, Supplementary Figure S2A shows confusion matrices of neural feature correlation coefficients between specific pairs of force trials and finger wiggling trials for each volitional state, averaged over 120 neural features with the highest signal-to-noise ratio, for five exemplary sessions and for all sessions averaged together. Correlation coefficients between pairs of force and wiggle trials were compared across volitional states, as well as within volitional states, to determine whether force-force and force-wiggle correlations were significantly different, and whether this difference was affected by volitional state. For all participant-grasp pairs except T8-power, correlation coefficients between force trials were greater during attempt than during observed and imagined force production (corrected p<0.05, t-test, Benjamini-Hochberg method). Additionally, within volitional states, correlation coefficients between pairs of force trials were often greater than correlation coefficients between force and wiggling trials, especially for attempted forces (corrected p<0.05, t-test, Benjamini-Hochberg method).

These trends are further illustrated in Supplementary Figure S2B, which shows distributions of force-force and force-wiggle correlation coefficients from all sessions for each volitional state. Here, both force-force and force-wiggle correlations rise as volitional state progresses from passive observation to active attempt. However, this change is significantly more pronounced for force-force correlations than for force-wiggle correlations (corrected p<0.05, Benjamini-Hochberg method). In other words, even though neural activity during force trials becomes increasingly correlated during attempt, force- and kinematic-related neural activity remains relatively less correlated across multiple volitional states.

Additionally, Supplementary Figure S3 shows session-average force embodiment (KFIQ) scores for all participants (0 = no feeling, 10 = feeling as intense as able-bodied execution of grasping force). Here, KFIQ scores vary with volitional state (attempt > imagine > observe, 2-way ANOVA). Additionally, while KFIQ scores are almost identical across forces within each volitional state, they are significantly lower during finger wiggling than they are for attempted forces in several participant-grasp pairs (p<0.05, paired-t-test, Benjamini-Hochberg correction). In other words, the degree to which participants kinesthetically emulate forces is strongest during attempted force, and is distinct from the degree to which they emulate forces during kinematic finger wiggling. Taken together, these results support the idea that neural activity during force trials encompassed modulation to kinetic parameters, rather than solely representing modulation to kinematic factors such as changes in hand posture.


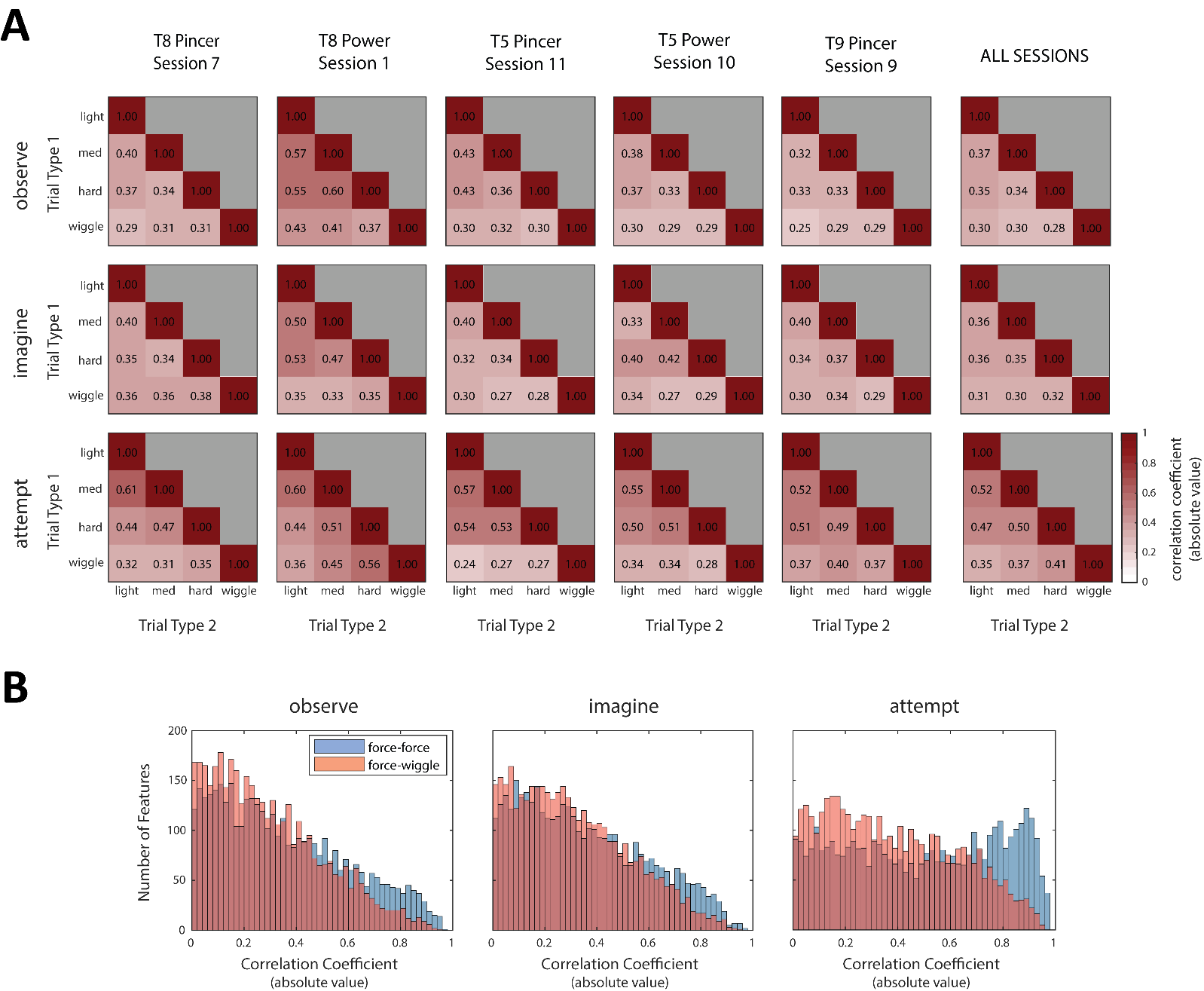


**Figure S4. Correlation between Kinetic and Kinematic Activity.** **A.** Heat maps of correlation coefficients between pairs of trial-averaged feature time courses, averaged over 120 features with highest signal-to-noise ratio for a representative session from each participant-grasp pair. Note that the last column shows session-averaged correlation coefficients. For nearly all sessions, correlations between attempted force and finger wiggle trials are smaller than correlations between two types of attempted force trials. **B.** Distributions of correlation coefficients between pairs of force trials (blue) and pairs of force and wiggle trials (orange) across all sessions. Note that during attempted trials, force trials are more correlated to each other than to finger wiggling trials.


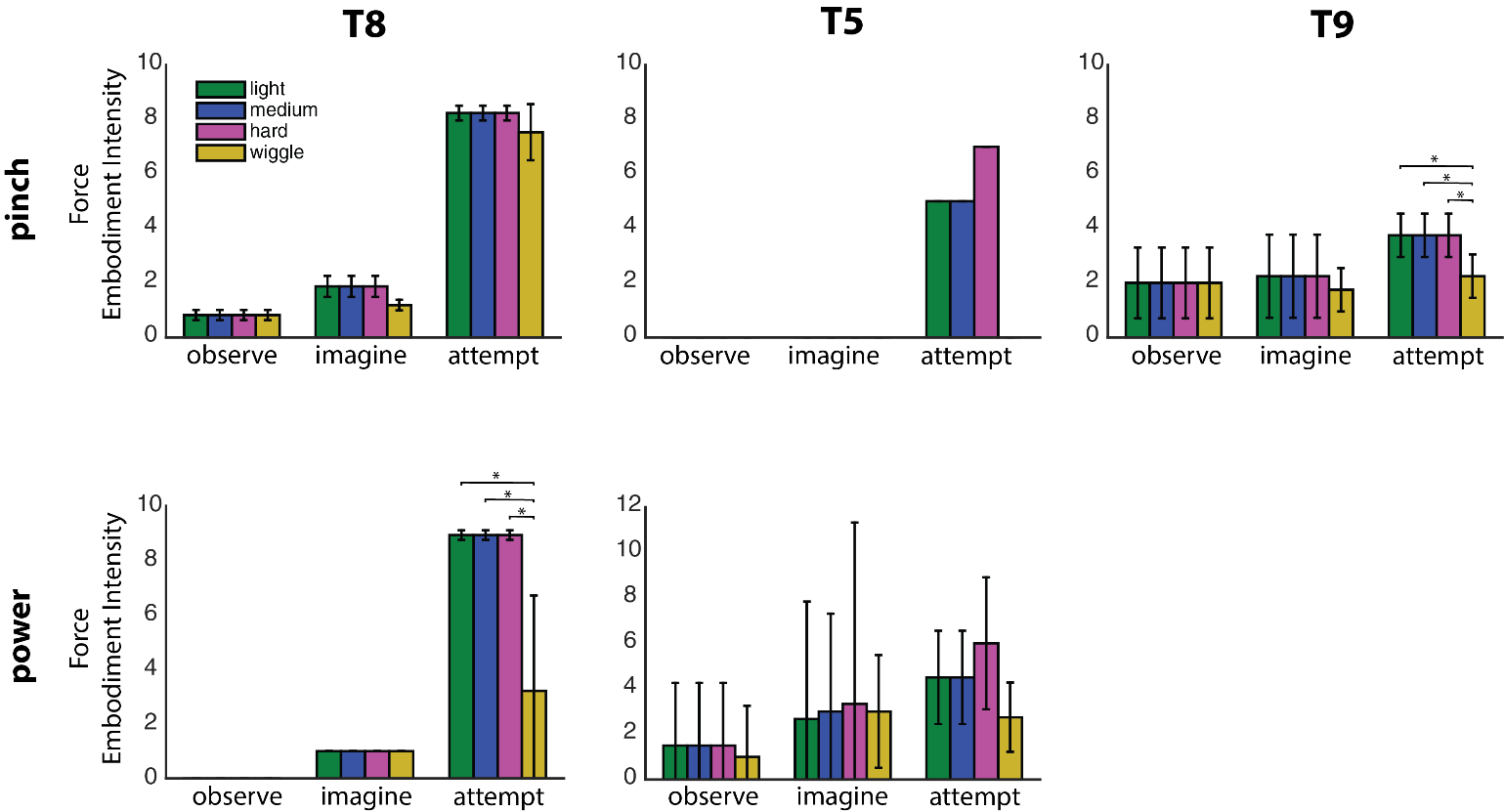


**Supplementary Figure S5:** **Session-averaged kinesthetic force imagery questionnaire (KFIQ) scores.** Participants rated on a scale of 0-10 how intensely they felt themselves producing light, medium, and hard forces during each volitional state and each grasp type (0 = no feeling, 10 = feeling as intense as able-bodied execution.) They were also asked to rate how intensely they emulated forces during finger wiggling trials. Scores were collected at the end of each experimental block in all sessions except Session 11 (T5 pincer), in which scores were recorded solely at the end of the session. Due to user error, participant T8 only reported accurate scores during sessions 5-8, so only these sessions are included in column 1. Also note that the absence of bars in T8 power and T5 pincer indicate KFIQ scores of 0 (T5 pincer, T8 power). Error bars indicate 95% confidence intervals. Asterisks indicate significant differences in KFIQ scores between trial types within a volitional state (p<0.05, paired t-test, Benjamini Hochberg correction). In all participants, force embodiment increases with volitional state. Additionally, for several participants and grasps, KFIQ scores during finger wiggling are significantly lower than KFIQ scores during attempted force production.

**Assessment of Tuning to Force and Volitional State**

Supplementary Figures S6 and S7 show average per-condition activity (PSTH) of hand-selected threshold crossing (TC) and spike band power (SBP) features in participants T5 and T9, respectively. As in participant T8 (Figure 2), these features were tuned to one of four marginalizations as evaluated by 2-way Welch-ANOVA analysis implemented on go-phase neural activity: *force only, volitional state only, both* force and volitional state, and an *interaction* between force and volitional state. Note that Welch-ANOVA detected no features with a significant (corrected p<0.05) interaction between force and volitional state in participant T9.

In addition, Supplementary Figures S8-S11 depict single unit and multiunit features extracted from four representative channels in participant T8. The multiunit features from these four channels, which include threshold crossing rate and spike band power, were tuned to force (S8, S10), volitional state (S9), both factors (S10, S11), and an interaction (S9, S11) between force and volitional state. Note that the first detected unit from each channel was often a “hash” unit (Todorova et al, 2014), which encompassed neural activity that could not be assigned to a “true” unit but nonetheless contained useful force or volitional state information. In the four representative channels depicted here, the first sorted unit from each channel closely matched the activity of their corresponding threshold crossing feature both visually and statistically. Additional sorted units also exhibited statistical trends that were consistent with their associated threshold crossing and spike band power features. For example, Supplementary Figure S11 shows that threshold crossing and spike band power features extracted from Channel 65 reflected the underlying activity of three single units. Specifically, the hash unit and Unit 2 were statistically tuned to an interaction between force and volitional state, as reflected in the activity of the threshold crossing feature; while Unit 1 was independently tuned to force and volitional state, as reflected by the activity of the spike band power feature. These data suggest that single sorted units and extracted multiunit features contained similar information in regards to force and volitional state, which in turn suggests that an analysis of sorted single units would yield similar results to those identified in the main text.

**
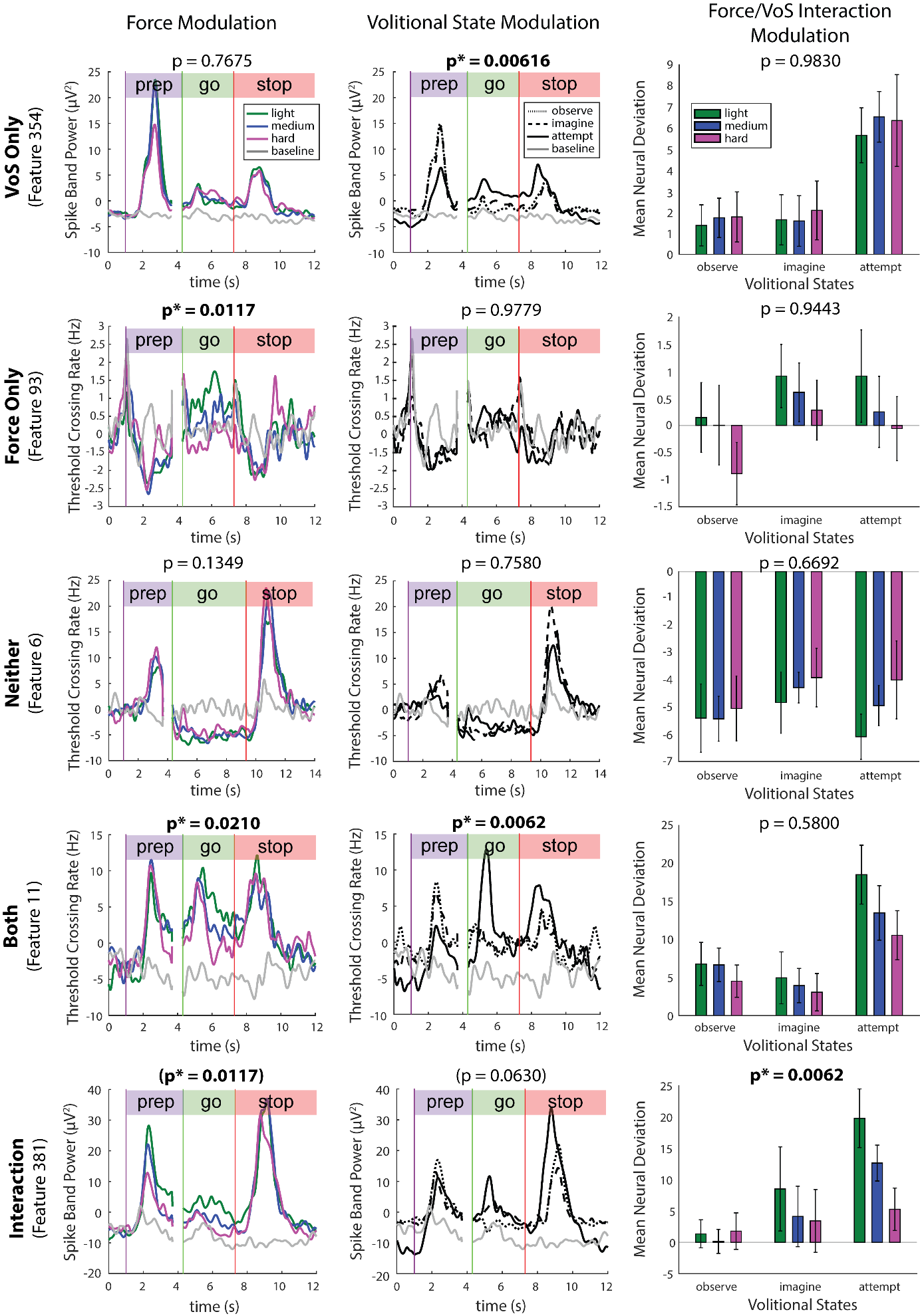
**

**Figure S6. Single features are tuned to force and volitional state in participant T5.** Rows show average PSTHs of five exemplary TC and SBP features that are tuned to force only (session 10), volitional state (VoS) only (session 10), and neither factor (session 11), both factors (session 10), and an interaction between force and volitional state (session 10) (2-way Welch ANOVA, corrected p<0.05, Benjamini-Hochberg method). Columns show neural activity averaged over all forces (column 1), neural activity averaged over all volitional (column 2), and normalized mean go-phase neural deviations from baseline activity during force trials within each force-VoS pair (column 3). Statistically significant p-values for force modulation, VoS modulation, and interaction are indicated with asterisks. Error bars indicate 95% confidence intervals.

**
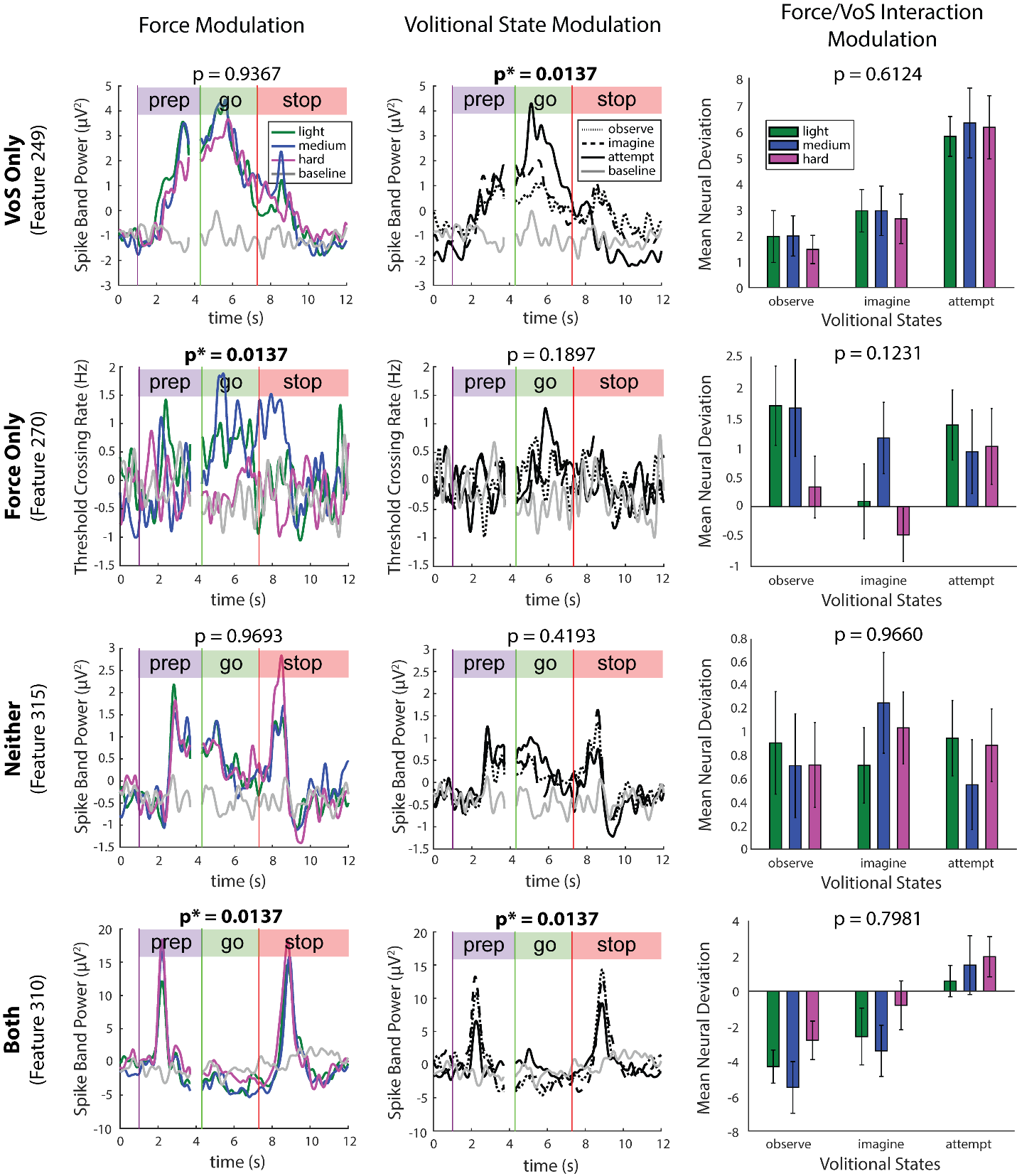
Figure S7. Single features are tuned to force and volitional state in participant T9.** Rows show average PSTHs of four exemplary TC and SBP features from Session 9 that are tuned to force only, volitional state (VoS) only, neither factor, and both factors (2-way Welch ANOVA, corrected p<0.05, Benjamini-Hochberg method). Note that 2-way Welch-ANOVA detected no features with a statistically significant interaction between force and volitional state. Columns show neural activity averaged over all forces (column 1), neural activity averaged over all volitional (column 2), and normalized mean neural deviations from baseline activity during force trials within each force-VoS pair (column 3). Statistically significant p-values for force modulation, VoS modulation, and interaction are indicated with asterisks. Error bars indicate 95% confidence intervals.

**
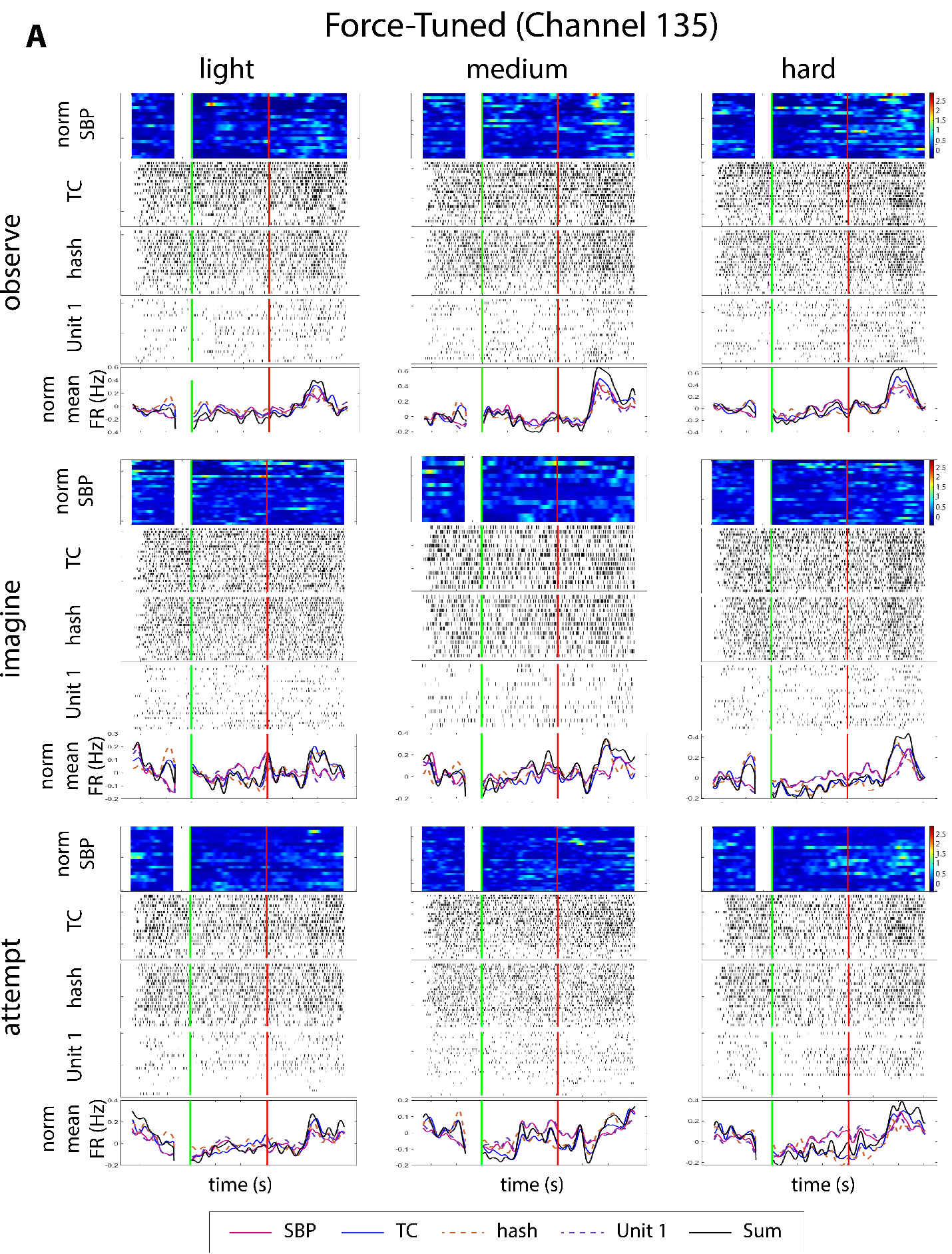

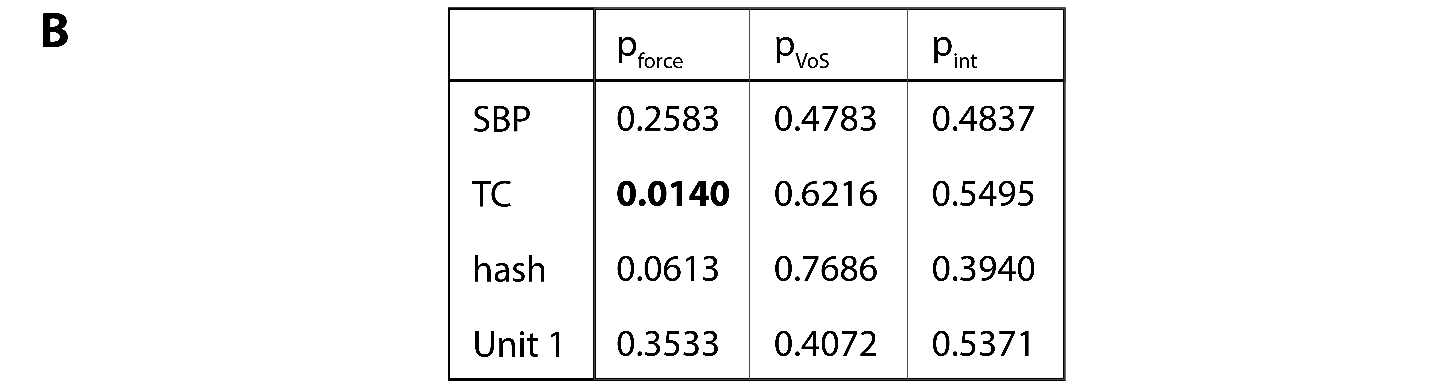
**

**Figure S8A.** Spike band power (SBP) data, threshold crossing (TC) rasters, and single unit rasters from a force-tuned channel. Each panel depicts SBP, TC and single unit activity extracted from channel 135 during Session 1, for individual volitional states (observe, imagine, attempt) and force levels (light, medium, hard). Within the last plot within each panel, activity across multiple single units was summed and visually compared to normalized, trial-averaged threshold crossing and spike band power activity for each volitional state and force level. Here, the active “go” phase of the trial occurred between the vertical lines. Note that the hash unit closely matches TC activity, while Unit 1 closely matches SBP activity. **B.** 2-way Welch ANOVA p values (p<0.05 in bold) for extracted SBP, TC, and single unit features. Note that the hash unit appears to exhibit force-related activity that approaches (but does not exceed) statistical significance.

**
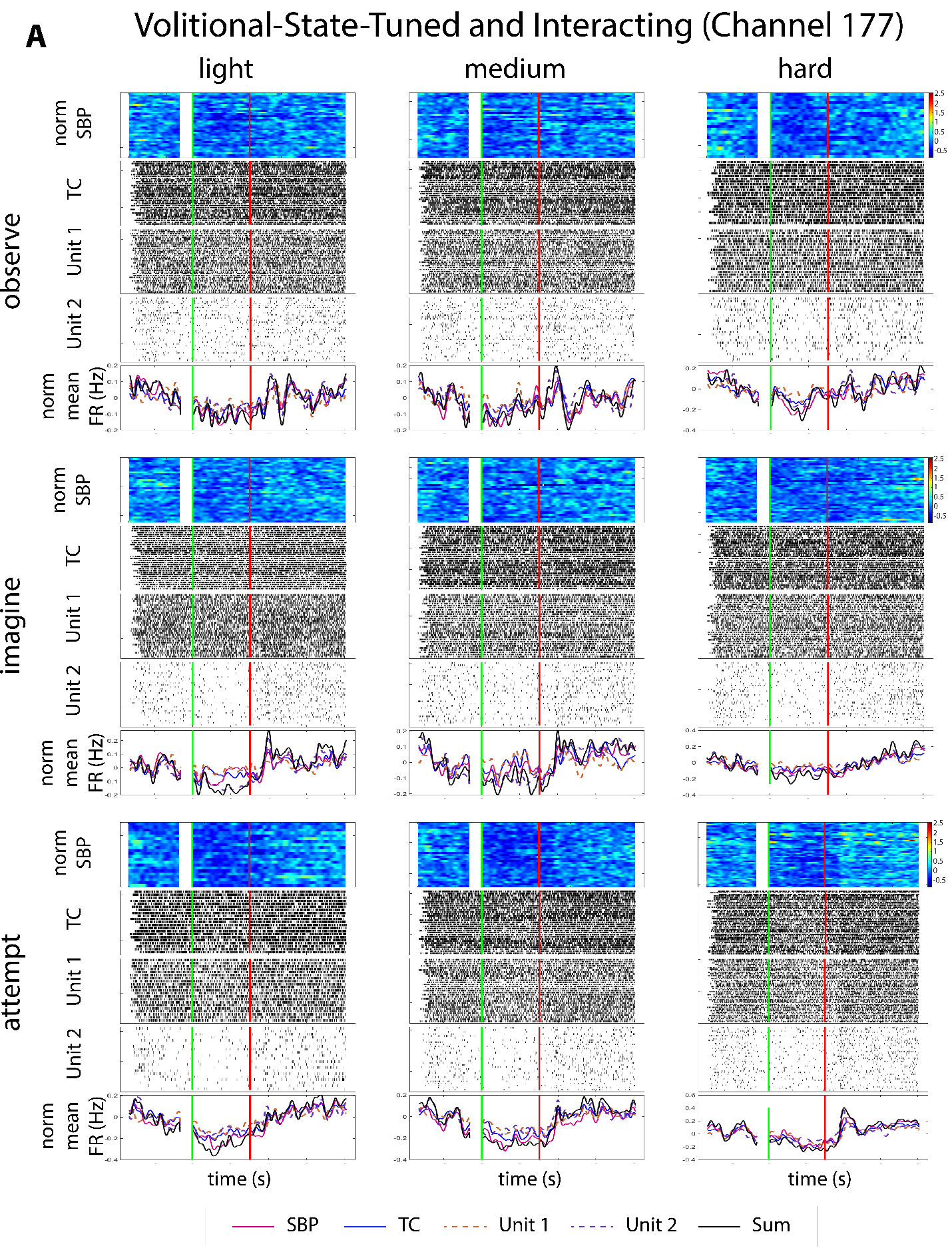

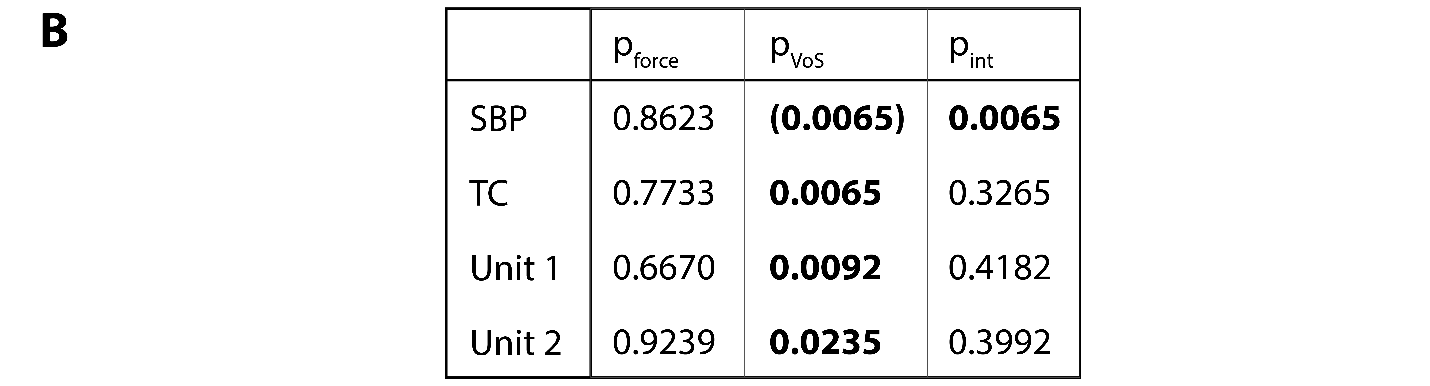
**

**Figure S9A.** Spike band power (SBP) data, threshold crossing (TC) rasters, and single unit rasters from a channel tuned to volitional state (TC, Unit 1, Unit 2) and to an interaction between force and volitional state (SBP). Each panel depicts single unit and multiunit features extracted from channel 177 during Session 4, for individual volitional states (observe, imagine, attempt) and force levels (light, medium, hard). Within the last plot within each panel, activity across multiple single units was summed and visually compared to normalized, trial-averaged threshold crossing and spike band power activity for each volitional state and force level. Here, the active “go” phase of the trial occurred between the vertical lines. **B.** 2-way Welch ANOVA p values (p<0.05 in bold) for extracted SBP, TC, and single unit features.

**
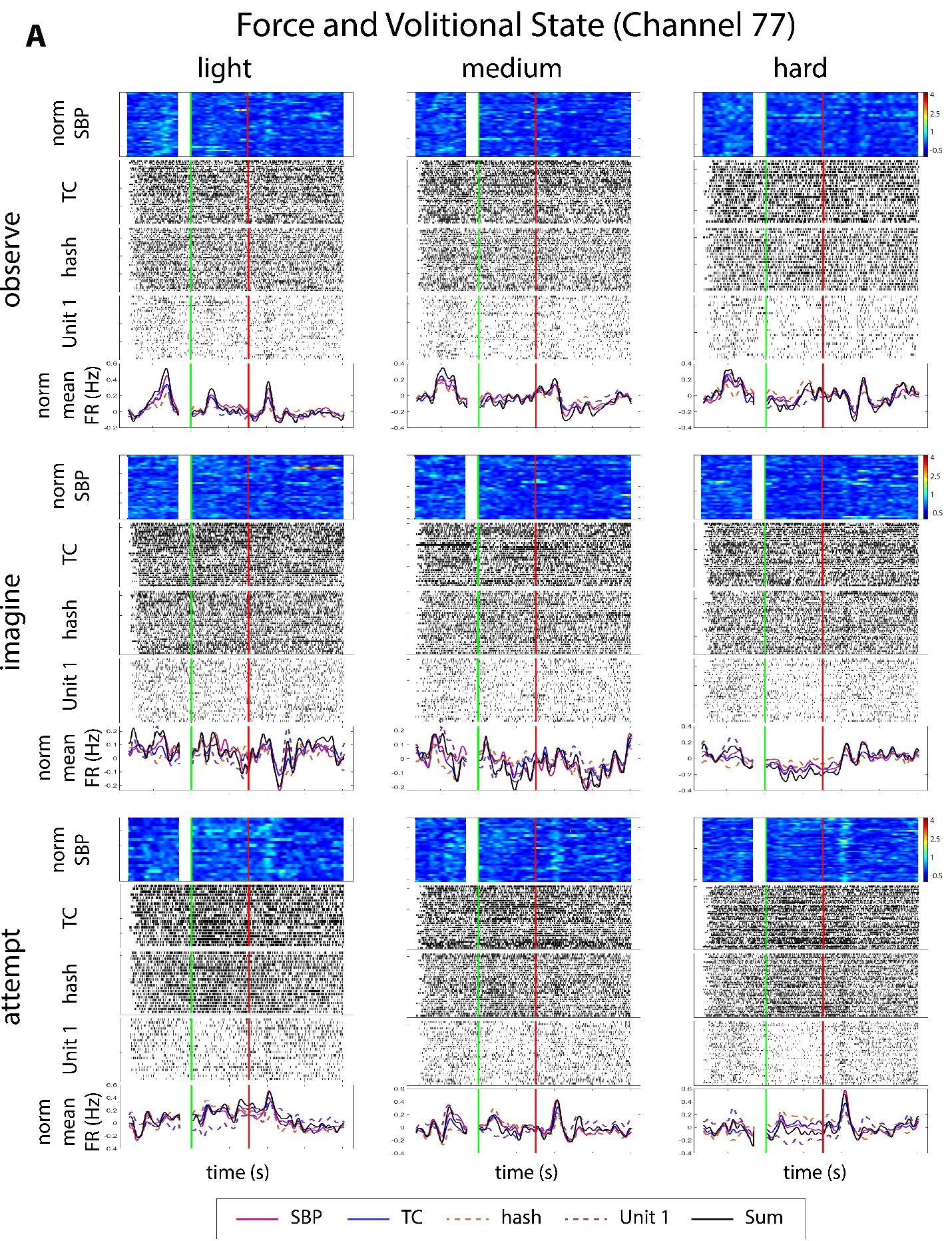

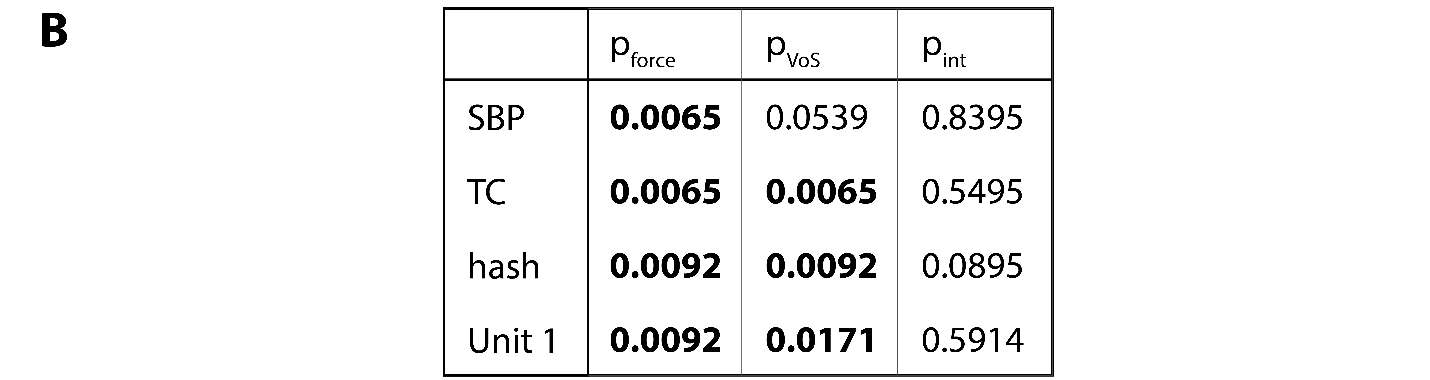
**

**Figure S10A.** Spike band power (SBP) data, threshold crossing (TC), and single unit rasters from a channel tuned to force (SBP) and independently tuned to both force and volitional state (TC, hash, Unit 1). Each panel depicts the activity of single unit and multiunit features extracted from channel 77 during Session 4, for individual volitional states (observe, imagine, attempt) and force levels (light, medium, hard). Within the last plot within each panel, activity across multiple single units was summed and visually compared to normalized, trial-averaged threshold crossing and spike band power activity for each volitional state and force level. Here, the active “go” phase of the trial occurred between the vertical lines. **B.** 2-way Welch ANOVA p values (p<0.05 in bold) for extracted SBP, TC, and single unit features. Note that the SBP feature exhibits statistically significant tuning only to force; however, its volitional state tuning also approaches (but does not exceed) statistical significance.

**
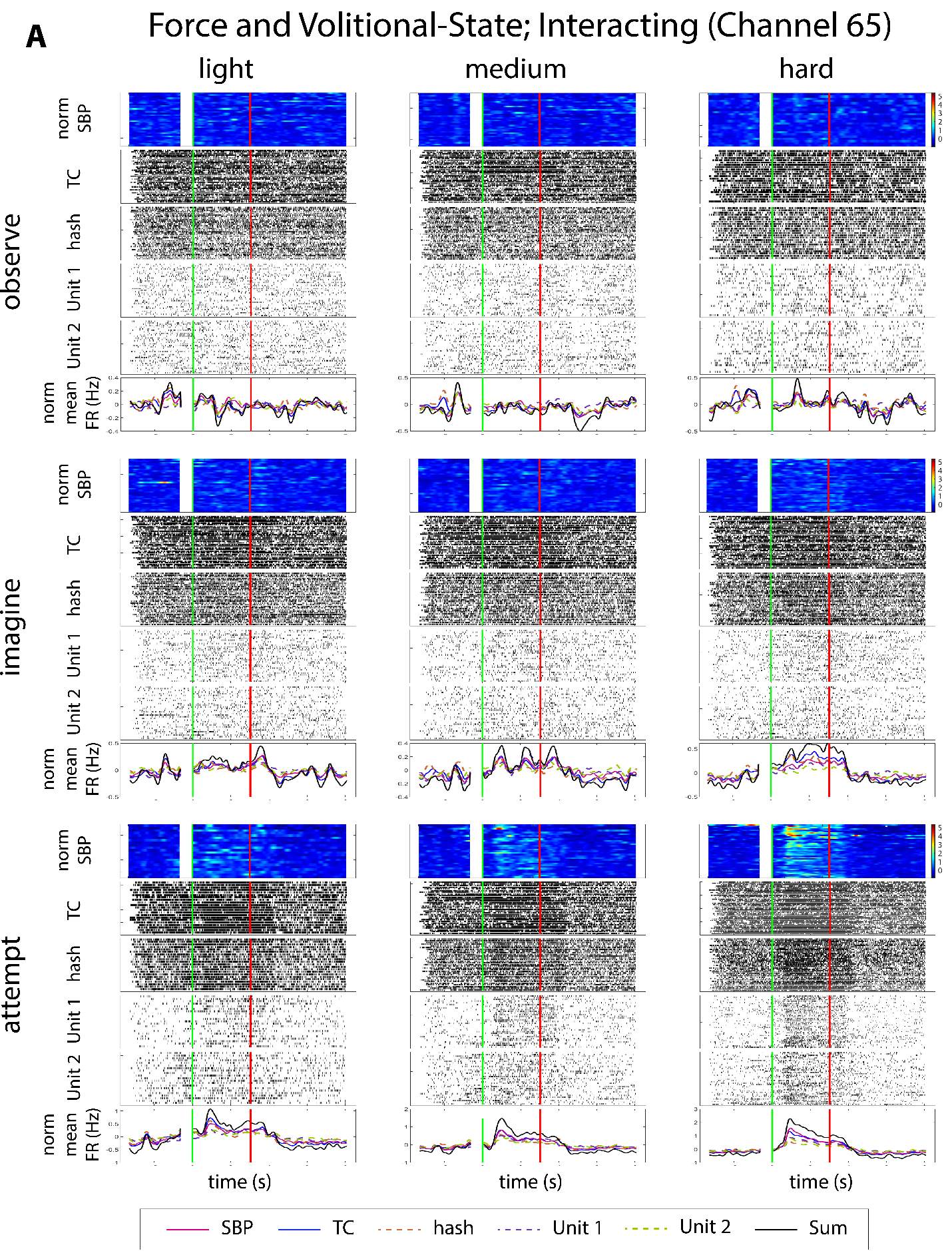

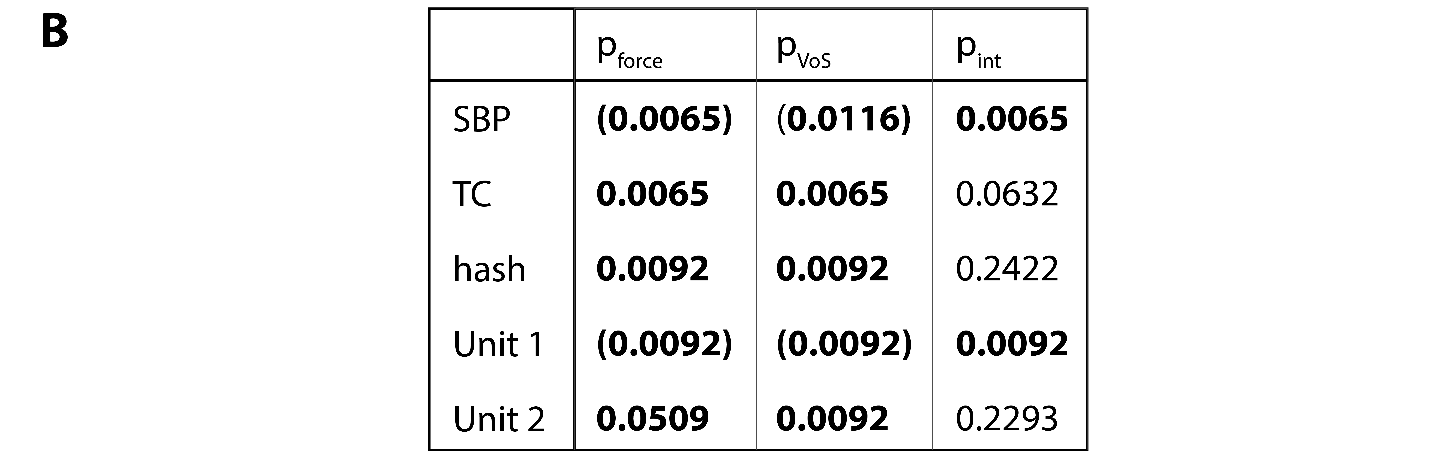
**

**Figure S11A.** Spike band power (SBP) data, threshold crossing (TC) rasters, and single unit rasters extracted from channel 65 during Session 4, for individual volitional states (observe, imagine, attempt) and force levels (light, medium, hard). Within the last plot within each panel, activity across multiple single units was summed (black trace) and visually compared to normalized, trial-averaged threshold crossing and spike band power activity for each volitional state and force level. Here, the active “go” phase of the trial occurred between the vertical lines. **B.** 2-way Welch ANOVA p values (p<0.05 in bold) for extracted SBP, TC, and single unit features. Here, the SBP feature is tuned to the interaction between force and volitional state, which appears to capture the activity of Unit 1. Likewise, the TC feature is independently tuned to force and volitional state and appears to reflect the trends exhibited by the hash unit and Unit 2.
